# Microstructural Evolution, Sigma Phase Morphology, and Localized Corrosion Behavior in UNS S32750 Superduplex Stainless Steel

**DOI:** 10.3390/ma19163413

**Published:** 2026-08-11

**Authors:** Priscila Sousa Nilo Mendes, Patricia Sousa Nilo Mendes Raider Leoni, Elivelton Alves Ferreira, Gláucio Soares da Fonseca

**Affiliations:** Graduate Program in Metallurgical Engineering, Federal Fluminense University, Volta Redonda 27255-125, RJ, Brazil; priscilanilomendes@gmail.com (P.S.N.M.); patysnm@gmail.com (P.S.N.M.R.L.); eliveltonalves@id.uff.br (E.A.F.)

**Keywords:** superduplex stainless steel, sigma phase, selective corrosion, sensitization, chromium depletion

## Abstract

The effects of sigma (σ) phase morphology and distribution on the corrosion behavior of superduplex stainless steel (SDSS) were investigated. The aim of this study was to establish the relationship among distinct σ-phase morphologies, chromium redistribution, and the resulting corrosion mechanisms using electrochemical and microstructural characterization techniques. Heat treatments at 700 °C and 800 °C produced distinct σ-phase morphologies and volume fractions. Microstructural characterization was performed using optical microscopy (OM) and scanning electron microscopy (SEM), and corrosion behavior was evaluated using open-circuit potential (OCP), cyclic polarization, and double-loop electrochemical potentiodynamic reactivation (DL-EPR). Statistical analysis of chromium (Cr) distribution was used to link microstructural evolution to corrosion susceptibility. Lamellar σ promotes selective attack between lamellae of σ and secondary austenite due to localized Cr depletion, whereas σ at grain boundaries causes sensitization and intergranular corrosion. Notably, the sample aged at 800 °C for 30 h did not exhibit pitting during cyclic polarization. However, it showed clear signs of sensitization and selective attack in the DL-EPR test, indicating that temperature-driven Cr partitioning increases Cr gradients around σ. These gradients, together with the morphology and fraction of σ, ultimately govern the transition between lamellar-selective corrosion and grain-boundary sensitization. Overall, the findings underscore the importance of combining microstructural and electrochemical assessments to accurately predict localized corrosion and sensitization in SDSS.

## 1. Introduction

Duplex and superduplex stainless steels (DSS and SDSS) are widely used in the chemical, petrochemical, and marine industries because of their excellent balance of mechanical strength and corrosion resistance, which stems from the stable presence of ferrite (α) and austenite (γ) phases [1,2,3,4,5]. Their exceptional resistance to localized corrosion in chloride-rich environments makes them a preferred choice over austenitic stainless steels in aggressive conditions [6,7,8].

However, the complex microstructure of SDSS is metastable at intermediate temperatures (600–950 °C), thereby promoting the precipitation of secondary phases, especially the intermetallic sigma (σ) phase [9,10]. The σ-phase is rich in Cr and Mo, leading to local depletion of these alloying elements in the surrounding matrix, thereby greatly reducing both toughness and corrosion resistance [11,12,13,14]. Its precipitation is therefore a key factor that limits the processing and service performance of SDSS components.

Different sigma-phase morphologies can form depending on the aging temperature and precipitation kinetics. The lamellar, or coral-like, morphology is often observed at lower temperatures [3,9,15]. It results from the eutectoid decomposition of ferrite into sigma and secondary austenite. Since the mechanism is diffusion-controlled, Cr- and Mo-rich sigma lamellae alternate with Ni-enriched secondary austenite. At higher temperatures, where Cr and Mo diffusion occur more rapidly, sigma particles coarsen and develop a blocky morphology [9]. Moreover, during prolonged aging, three distinct morphologies can coexist within the microstructure: coral-like sigma, sigma decorating grain boundaries, and blocky sigma within ferritic structures [16].

The negative impact of sigma on corrosion resistance has been extensively documented, highlighting increased susceptibility to pitting and intergranular corrosion [14,17,18]. Studies using techniques such as double-loop electrochemical potentiodynamic reactivation (DL-EPR) and potentiodynamic polarization have shown that sensitization and localized attack increase with increasing σ-phase content [19,20,21]. However, most research focuses on the effects of sigma volume fraction, while the specific role of sigma phase morphology in corrosion mechanisms remains less well understood.

Recent studies have combined electrochemical testing with microstructural characterization to evaluate corrosion behavior in duplex and superduplex stainless steels. For example, Pitacco et al. [20] employed DL-EPR, along with detailed microstructural analysis, to assess sensitization and localized corrosion. Their approach provided valuable insights into phase-related corrosion susceptibility. Similarly, Breda et al. [21] investigated the effect of cold deformation on pitting resistance using electrochemical testing, highlighting performance variations across different processing conditions. Although these studies significantly contribute to understanding corrosion mechanisms, the specific role of distinct sigma-phase morphologies—such as coral-like, blocky, or grain-boundary precipitation—in determining the type of corrosion attack has not been systematically clarified.

Hosseini et al. [22] investigated the effect of sigma phase morphology on property degradation in a superduplex stainless steel (SDSS) subjected to arc heat treatment. They demonstrated that localized corrosion occurred in ferrite and secondary austenite near the coral-like sigma phase. Although sensitization screening tests showed that regions containing sigma phase were more affected, detailed microstructural evidence—such as SEM images highlighting the most affected areas—was not provided. These findings nevertheless underscore the importance of sigma-phase morphology in the corrosion behavior of SDSS.

Liu et al. [23] explored the evolution of the sigma phase and its impact on the corrosion resistance of ASTM A890 3A duplex stainless steel. They observed a clear correlation between the sigma-phase volume fraction and the alloy’s corrosion behavior. Aging treatments were conducted at 750 °C for various durations. As the σ-phase content increased from 0 to 0.65%, the passive current density increased while the pitting potential decreased. When the σ-phase fraction ranged from 0.65% to 19.07%, the passive region became narrower and less stable. The shift from passive to active corrosion behavior occurred after approximately 48 h of aging, when about 19.07% of the σ phase was present, and the passive region had nearly vanished. The uniformity of σ-phase distribution was also found to influence the corrosion resistance.

After 48 h of aging, two morphologies of σ-phase precipitation were identified: a uniform form and a net-like structure distribution. With longer aging times (72 and 96 h), the σ phase became more evenly distributed. Overall, the decline in DSS corrosion resistance was closely linked to the extent and morphology of σ-phase precipitation.

Once again, the study by Liu et al. [23] was particularly interesting because it examined the relationship between the amount of sigma phase formed and corrosion resistance. However, the details of the sigma-phase morphology were shown only for the 48 h-aged sample. In comparison, the microstructure revealing pit formation and intergranular corrosion was observed only in the sample aged for 72 h. Therefore, a more detailed microstructural analysis was missing.

Sampaio et al. [19,24] showed that the sigma phase exhibited two morphologies—lamellar and block—and that each of them resulted in distinct electrochemical responses. They observed significant changes in the linear sweep voltammetry (LSV) curves as a function of sigma-phase morphology. They conclude that morphology is governed by sigma-phase precipitation kinetics and can help identify the properties most affected by its formation.

In this context, linking the microstructural evolution of the sigma phase—including coral-like, blocky, and grain-boundary morphologies—to electrochemical behavior is essential to clarifying the relationship between precipitation features and corrosion resistance in SDSS. This approach provides a clearer understanding of the conditions that lead to localized versus selective corrosion, thereby aiding material design and lifetime forecasting.

Previous studies by our research group investigated the precipitation kinetics, formation mechanisms, and microstructural evolution of the sigma phase in duplex and superduplex stainless steels [1,25]. These studies established the metallurgical basis for understanding sigma-phase formation but did not examine its electrochemical behavior or corrosion mechanisms. Building on this foundation, the present work focuses on correlating distinct sigma-phase morphologies with localized corrosion behavior through electrochemical techniques and detailed microstructural characterization.

Therefore, the aim of this study is to establish a detailed and systematic correlation between sigma-phase morphology (coral-like, blocky, and grain-boundary precipitation) and the resulting corrosion mechanisms in UNS S32750 superduplex stainless steel. Unlike previous corrosion studies in the literature, which have primarily focused on the sigma-phase volume fraction, the present work combines quantitative sigma-phase analysis with extensive compositional mapping (461 EDS measurement points) and electrochemical techniques, including OCP, potentiodynamic polarization, and DL-EPR.

This integrated microstructural–electrochemical approach offers a more comprehensive understanding of how specific sigma morphologies influence chromium-depletion patterns and the transition between selective and localized corrosion. By explicitly linking morphology, compositional heterogeneity, and electrochemical response, this study advances a more mechanistic interpretation of corrosion degradation in SDSS.

## 2. Materials and Methods

This section describes procedures for preparing, characterizing, and evaluating the electrochemical behavior of superduplex stainless steel, with a focus on the effects of sigma-phase precipitation. Heat-treated samples exhibiting sigma-phase volume fractions (*V_Vσ_*) ranging from 0.02 to 0.36 were selected to directly compare their corrosion resistance and sensitization behavior.

The investigated material was a commercial UNS S32750 superduplex stainless steel. Its chemical composition is presented in Table 1.

The investigated material was a commercial hot-rolled UNS S32750 superduplex stainless steel supplied by Aperam South America (Timóteo, MG, Brazil). Following [25], the steel was heat-treated at 700 °C and 800 °C for different holding times to investigate the kinetics of sigma-phase precipitation.

Samples treated at 700 °C for 2 h, 12 h, and 50 h, and at 800 °C for 1 h, 7.5 h, and 30 h were selected to achieve low, medium, and high sigma-phase levels, respectively. Because sigma-phase precipitation kinetics are strongly temperature dependent, different holding times were required at 700 °C and 800 °C to obtain comparable stages of precipitation. These heat-treatment conditions were selected to produce different sigma-phase volume fractions (*V_Vσ_*), ranging from low to high levels of precipitation, for subsequent electrochemical evaluation.

The aging temperatures and times were selected based on previous studies by our research group on the precipitation kinetics of sigma phase in superduplex stainless steels [1,25]. These studies investigated the nucleation and growth of the σ-phase as a function of temperature and time and identified conditions that yield different σ-phase volume fractions. Therefore, the selected aging treatments were designed to produce microstructures with low, intermediate, and high fractions of the σ phase to evaluate their influence on corrosion behavior.

These temperatures fall within the range where sigma-phase precipitation is most pronounced in duplex and superduplex stainless steels, as reported in TTT diagrams for this class of alloys [1]. Although they do not represent the typical service temperatures of UNS S32750, they may be locally reached during welding thermal cycles or under improper heat treatment and slow-cooling conditions, promoting sigma-phase precipitation. Therefore, they were selected as controlled laboratory aging temperatures to produce different sigma-phase morphologies and volume fractions for the electrochemical evaluation in this study.

### 2.1. Sample Preparation

The samples were ground with silicon carbide (SiC) abrasive papers (Aropol 2V, Arotec, Cotia, Brazil) with grit sizes from 120 to 2500 mesh under continuous water lubrication. Subsequently, the specimens were polished with diamond suspensions of 6 μm, 3 μm, and 1 μm on a felt polishing cloth with a lubricant for ferrous alloys (AUTOMET 250, Buehler, Lake Bluff, IL, USA). After polishing, the samples were rinsed with distilled water, cleaned with isopropyl alcohol, and dried with warm air. The microstructure was then revealed by immersion for 90 s in a modified Behara solution containing 20 mL HCl, 80 mL of distilled water, and 0.3 g of potassium metabisulfite (K_2_S_2_O_5_).

### 2.2. Microstructural Characterization

The Olympus BX51M optical microscope (Olympus, Tokyo, Japan), equipped with an SC30 digital camera, captured five micrographs per sample using Stream Basic software (version 8.1, Olympus, Tokyo, Japan). ImageJ (version 1.42q, National Institutes of Health, Bethesda, MD, USA) was used for quantitative analysis. Sigma-phase volume fractions (*V_Vσ_*) were calculated using conventional point-counting metallography [26]. In summary, *V_Vσ_* = *P_P_*, where *P_P_* represents the ratio of points on the sigma phase to the total number of points applied.

Based on the quantitative metallographic measurements, the selected aging conditions produced *V_Vσ_* values of approximately 0.02, 0.17, and 0.36 after aging at 700 °C for 2, 12, and 50 h, respectively, and 0.23, 0.25, and 0.33 after aging at 800 °C for 1, 7.5, and 30 h, respectively. Therefore, these conditions were considered representative of low, intermediate, and high sigma-phase contents for the electrochemical evaluation.

A detailed investigation of sigma phase formation and identification using specific metallographic procedures was previously reported by Fonseca et al. [25]. In this work, the precipitation kinetics and microstructural evolution of the σ phase in this alloy were systematically analyzed.

### 2.3. Electrochemical Tests

#### 2.3.1. Open-Circuit Potential (OCP) and Cyclic Polarization (CP)

A Tait-type electrochemical cell with an electrolyte reservoir and three electrodes was used. The setup included a reference electrode (Ag|AgCl|KCl_sat), a working electrode (AISI UNS S32750 stainless steel), and a platinum counter electrode. The working electrode was circular (1 cm^2^) and defined by the opening of the electrochemical cell, ensuring that the specimen borders were not exposed to the electrolyte. Working electrodes were ground before testing.

Electrochemical measurements were conducted in a solution containing 2 mol/L H_2_SO_4_ and 1 mol/L HCl [27]. Mixed H_2_SO_4_/HCl solutions have been widely used to investigate selective dissolution in duplex stainless steels, since ferrite and austenite phases may exhibit different dissolution behavior depending on the applied potential. This environment allows the identification of active–passive transitions and preferential phase dissolution. In the present work, this electrolyte was used to evaluate the electrochemical behavior of microstructures containing sigma phase. The solution was prepared with deionized water and used at room temperature. A fresh solution was used for each test. Each experiment was repeated five times to ensure reproducibility.

The tests followed a two-stage process. In the first stage, open-circuit potential (OCP) measurements determined the corrosion potential, which naturally stabilizes at the metal–electrolyte interface in the absence of an external current. After OCP stabilized, the second stage involved cyclic polarization (CP).

OCP values were recorded with a portable PalmSens EmStat3+ potentiostat (PalmSens BV, Houten, The Netherlands). PSTrace software (version 5.11) (PalmSens BV, Houten, The Netherlands) generated the curves. The electrodes remained immersed in the electrolyte for 1800 s.

For CP, the scan began 0.2 V below the corrosion potential and extended 1.5 V above it before reversing. The scan rate was 1 mV s^−1^, and each test lasted approximately 50 min.

After testing, samples were rinsed with distilled water and isopropyl alcohol. Surface morphology was examined using a Zeiss EVO MA10 scanning electron microscope (Carl Zeiss, Stuttgart, Germany) equipped with a LaB_6_ filament.

#### 2.3.2. Double-Loop Electrochemical Potentiodynamic Reactivation (DL-EPR)

The degree of sensitization (DOS) was assessed using the DL–EPR test. The working electrode was immersed in a solution containing 2 mol/L H_2_SO_4_ with 1.5 mol/L HCl [17].

Different electrolytes were employed because the electrochemical techniques used in this study pursue different objectives. The H_2_SO_4_/HCl solution adopted for the OCP and cyclic polarization tests was selected to evaluate passivation behavior and localized corrosion, whereas the electrolyte used for the DL-EPR test was selected according to the established DL-EPR methodology for assessing sensitization by promoting the selective reactivation of chromium-depleted regions adjacent to sigma-phase precipitates [17].

Samples were anodically polarized from the corrosion potential to +400 mV to reach the passive state (activation). The scan direction was then reversed, and samples were polarized cathodically back to the corrosion potential (reactivation). Both scans employed a scan rate of 1.5 mV s^−1^ [17].

Activation current density peaks (iₐ) and reactivation current density peaks (iᵣ) were measured. DOS was calculated as the ratio of maximum reactivation current density to maximum activation current density. (DOS = iᵣ/iₐ) [28]. DL-EPR tests were performed in quintuplicate for each condition. The activation (iₐ) and reactivation (iᵣ) current densities were defined as the maximum current density values in the forward and reverse scans, respectively. This criterion was consistently applied to all measurements. The reported curves correspond to the average response.

After testing, samples were rinsed with distilled water and isopropyl alcohol. Surface morphology was examined again using the Zeiss EVO MA10 SEM. Chromium distribution was evaluated using energy-dispersive X-ray spectroscopy (EDS). For each selected condition, several line scans were drawn across the corrosion-affected regions after the DL-EPR test. The EDS software (version 3.0) automatically generated point analyses along these lines, resulting in a total of 461 measurement points for each condition. This procedure provided a statistically representative assessment of chromium distribution and possible chromium depletion associated with the corrosion process.

## 3. Results and Discussion

### 3.1. Initial Microstructural Characterization

**Figure 1:**
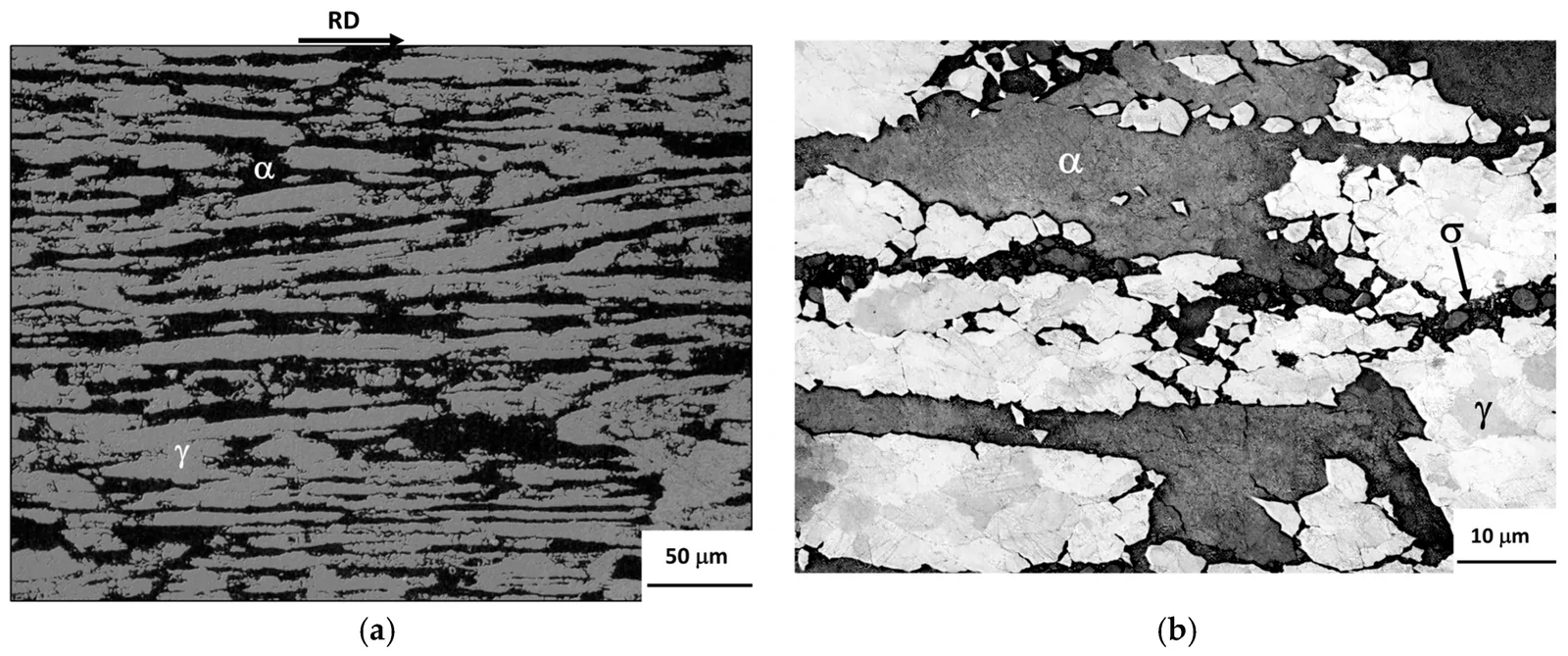

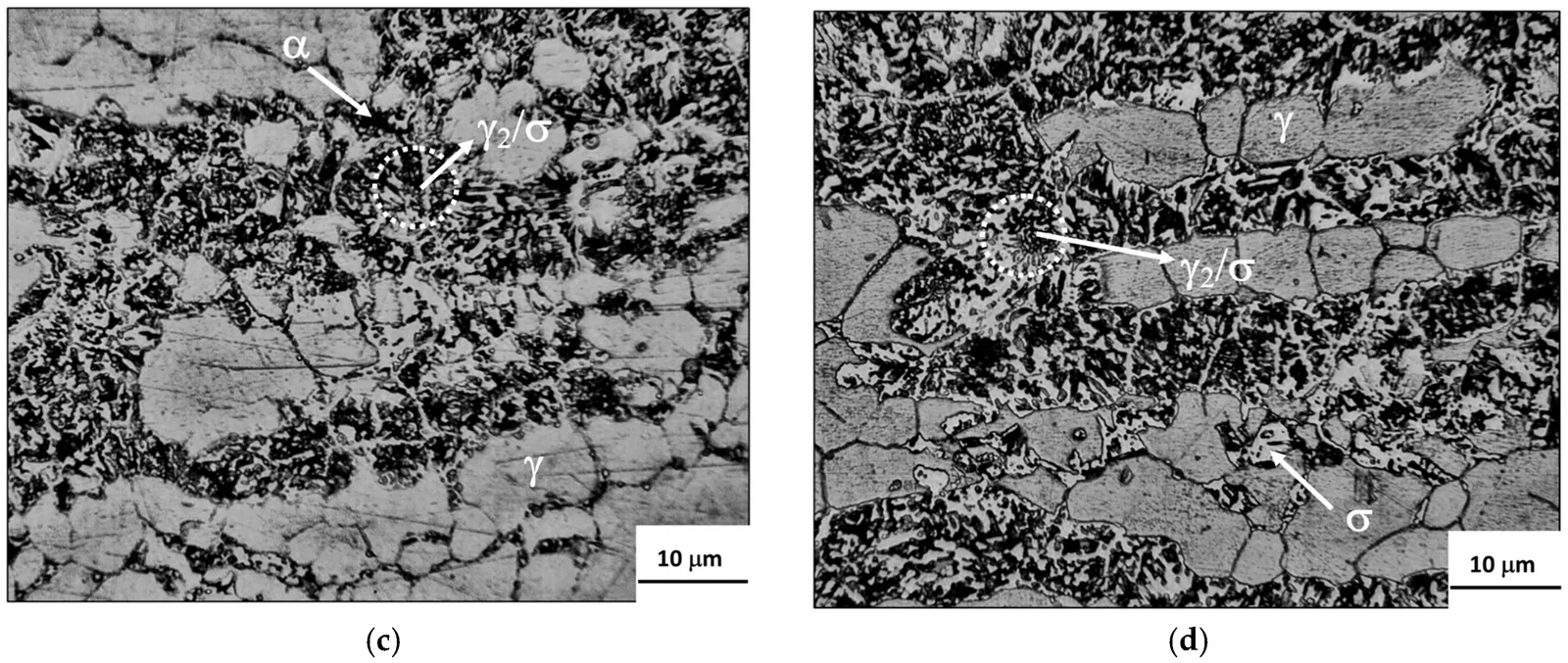
(**a**) Micrograph of the as-received sample (AR), showing α-ferrite (dark) and γ-austenite (light). The elongated grains of both phases are visible in the rolling direction (RD). (**b**) Micrograph of the sample treated at 700 °C for 2 h, showing α, γ, sigma phase σ (at some grain boundaries). (**c**) Micrograph of the sample treated at 700 °C for 12 h, showing α, γ, secondary austenite (γ_2_), and sigma (σ) in lamellae. (**d**) Micrograph of the sample treated at 700 °C for 50 h showing α, γ, secondary austenite (γ_2_), and sigma (σ) in lamellae.

Figure 1a displays the microstructure of the as-received superduplex stainless steel sample, where ferrite (α) appears as the darkest phase and austenite (γ) as the lightest, as commonly described in the literature [1,3,4,25]. The initial fractions of austenite and ferrite are 0.53 and 0.47, respectively. Figure 1b shows the sample treated at 700 °C for 2 h. The sigma phase volume fraction (*V_Vσ_*) measured under the conditions in Figure 1b was 0.02.

After 12 h of heat treatment (Figure 1c), the steel exhibits increased sigma (σ) and reduced ferrite (α). The microstructure in Figure 1c illustrates the formation of sigma (σ) in lamellae, likely interspersed with austenite, indicating that sigma forms through the eutectoid decomposition of ferrite (α → σ + γ_2_) [9,15,29]. The sigma phase volume fraction (*Vv_σ_*) measured in the condition shown in Figure 1c was 0.17.

As heat-treatment duration increases, the sigma phase grows as ferrite is consumed, becoming more prominent at 50 h (Figure 1d). The sigma phase volume fraction (*V_Vσ_*) measured under the conditions shown in Figure 1d was 0.36. As depicted in Figure 1d, the sigma phase also formed along certain grain boundaries, partially decorating them.

According to Pohl and coauthors [9], different morphologies of the sigma phase can appear depending on the working temperature. During lamellar decomposition, sigma grows cooperatively, forming lamellae of the sigma and secondary austenite phases within the ferrite structure, utilizing the chromium released by the ferrite. This process occurs in materials exposed to temperatures between 700 °C and 800 °C. Furthermore, Pohl and coauthors state that in the material aged between 750 °C and 800 °C, a σ phase with a “coral-like” morphology forms, characterized by a coral-like structure with smaller sigma (σ) phase plates distributed within a network.

**Figure 2:**
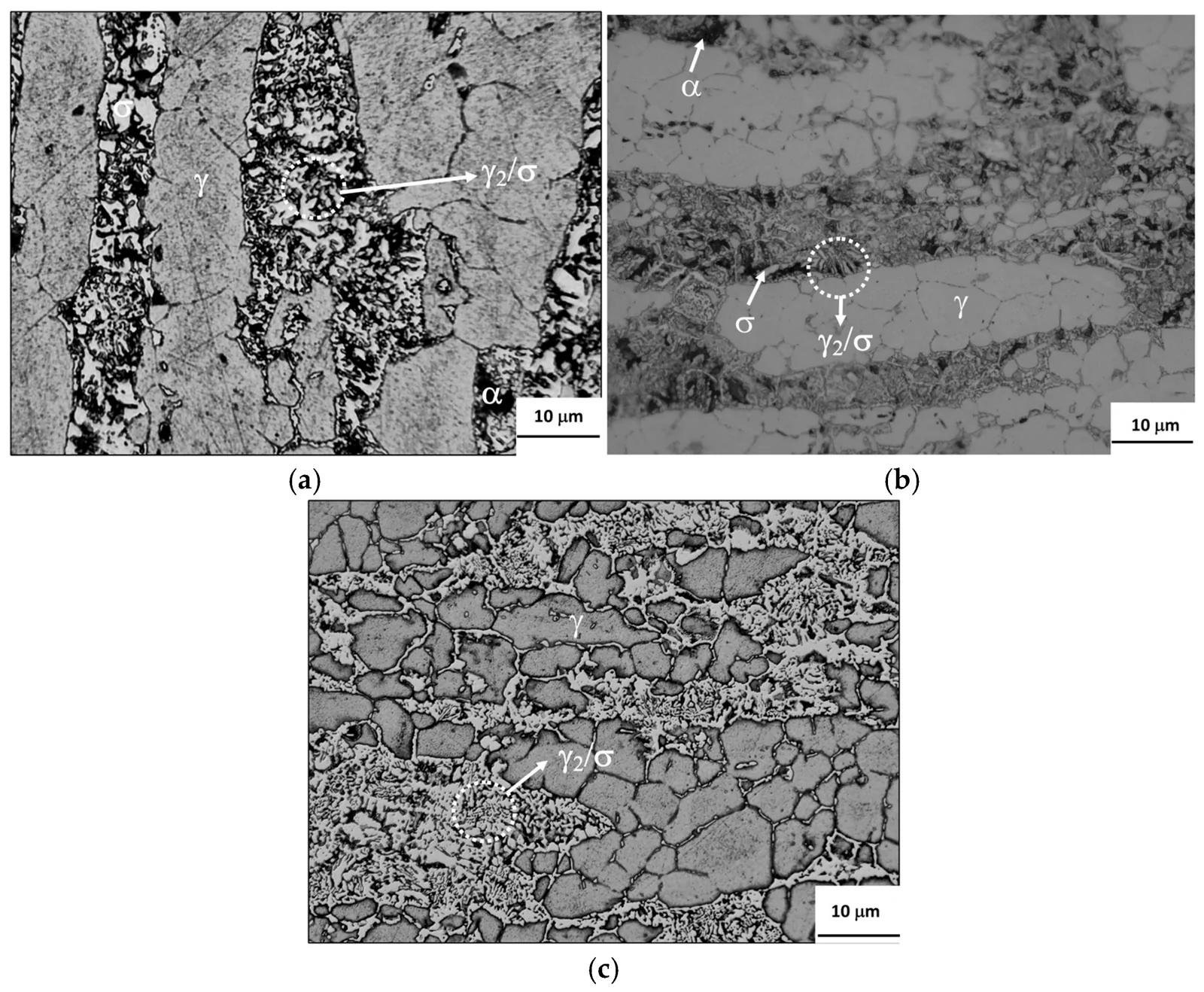
(**a**) Micrograph of the sample treated at 800 °C for 1 h, showing α-ferrite (dark) and γ-austenite (light brown), with the σ lighter phase and γ_2_/σ in lamellae. (**b**) Micrograph of the sample treated at 800 °C for 7.5 h, showing α-ferrite (dark) and γ-austenite (light brown), with the σ lighter phase and γ_2_/σ in lamellae. (**c**) Micrograph of the sample treated at 800 °C for 30 h showing γ-austenite (light brown), with the σ lighter phase and γ_2_/s in lamellae.

Figure 2a shows the microstructure of the sample treated at 800 °C for 1 h. It features the sigma phase with a coral-like shape, indicating eutectoid decomposition of ferrite (α → σ + γ_2_), which would produce intercalated sigma and secondary austenite. There are also larger sigma formations between the ferrite and austenite grains, likely representing other possible sigma formation mechanisms such as divorced eutectoid decomposition (blocky) [9]. The sigma volume fraction was 0.23. Figure 2b shows the microstructure of the sample treated at 800 °C for 7.5 h, which is similar to that shown in Figure 2a, with a sigma volume fraction of 0.25.

Finally, the microstructure of the sample treated at 800 °C for 30 h is shown in Figure 2c. The sigma morphology varies in this condition. There is a massive sigma phase, as well as a sigma phase intercalated with secondary austenite. Additionally, the sigma phase forms extensively along austenite grain boundaries, decorating nearly all of them. The sigma phase volume fraction under this condition was 0.33.

### 3.2. Electrochemical Testing

The curves obtained from open-circuit potential (OCP) and polarization curves (PC) are shown below. Also shown are the curves from the double-loop electrochemical potentiodynamic reactivation (DL-EPR) test used to calculate the degree of sample sensitization (DOS). Each curve is accompanied by SEM micrographs of the tested regions, aiding in electrochemical characterization.

#### 3.2.1. Open-Circuit Potential

Potential-time (E × t) monitoring tests were performed in the solution for 1800 s of immersion before polarization (CP) to evaluate the material’s corrosion behavior in the electrolyte. In this test, the anodic and cathodic electrochemical reactions occur spontaneously and simultaneously. No external current is applied, and the aim is to track the sample’s potential (V) over time.

**Figure 3:**
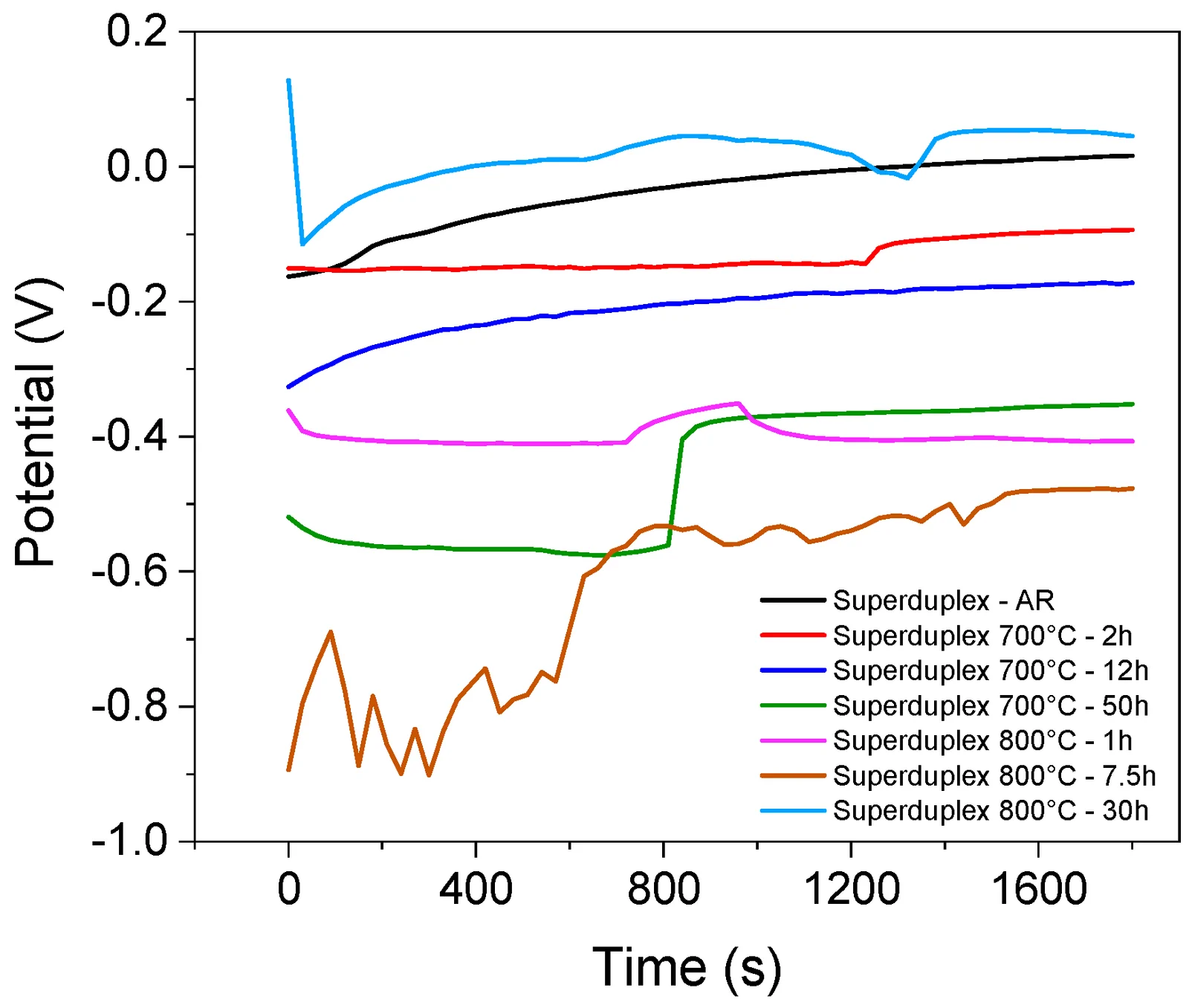
Open-circuit potential curves of superduplex stainless steel in 2H_2_SO_4_ + 1HCl solution for 1800 s at 25 °C.

Figure 3 shows the potential versus time curves obtained from this test for superduplex stainless steels under different treatment conditions, as presented in item 3.1. According to Tait [30], the direction the curve follows during the test is related to how the material reacts upon contact with the electrolyte.

For superduplex stainless steel, the curves exhibit a behavior similar to the stationary phase of the open-circuit potential (Eocp) already around the thousandth second.

The progressive increase in Eocp suggests the formation and stabilization of a protective passive film, as commonly observed in stainless steels [6].

The initial oscillatory behavior of the open-circuit potential for the 800 °C/7.5 h sample—characterized by repeated increases and decreases up to approximately 1000 s—indicates metastable pitting, which involves transient local breakdown and rapid repassivation of the passive film. This phenomenon, commonly observed in stainless steels [31], results from localized anodic dissolution at microstructural heterogeneities (such as σ-phase precipitates or compositional inhomogeneities), followed by the local reformation of a chromium-enriched oxide. The gradual decline in oscillations and the stabilization of Eocp after about 1000 s suggest the formation of a more stable passive state or the exhaustion of the most active sites, thereby reducing further metastable events.

The average open-circuit potential (Eocp) values for various treatment conditions are presented in Table 2. According to Sedriks [6], higher Eocp values indicate greater resistance to metal corrosion, a claim that other testing methods should confirm.

Although sigma-phase precipitation generally shifts the OCP toward more negative values [32], the present results indicate that the open-circuit potential is not controlled exclusively by the sigma-phase volume fraction. The specimens aged at 800 °C for 1 h and 7.5 h exhibited more negative OCP values than the specimen aged at 700 °C for 50 h, despite their lower sigma-phase volume fractions. This behavior suggests that these conditions may be associated with more electrochemically active Cr-depleted regions surrounding the precipitated phases, indicating that the severity and distribution of local Cr depletion, rather than the overall sigma-phase fraction alone, play an important role in determining the OCP. This interpretation is consistent with previous studies showing that corrosion behavior depends not only on the amount of sigma phase but also on its morphology and chromium redistribution during aging [18].

It is interesting to note from Figure 3 and Table 2 that the AR sample exhibits a higher Eocp than the samples containing the σ phase. An exception is the specimen aged at 800 °C for 30 h, which—despite presenting a high σ-phase volume fraction (0.33)—shows a more noble Eocp than the other aged conditions. This behavior suggests that prolonged aging at 800 °C for 30 h promoted a more homogeneous redistribution of chromium between the sigma phase and the surrounding matrix than aging at 700 °C [17]. Previous compositional analyses by EDS in Ref. [1] showed that the sigma-phase composition under these conditions was closer to the equilibrium values predicted by Thermo-Calc simulations, indicating a more homogeneous partitioning of alloying elements between phases.

#### 3.2.2. Potentiodynamic Polarization Tests

**Figure 4:**
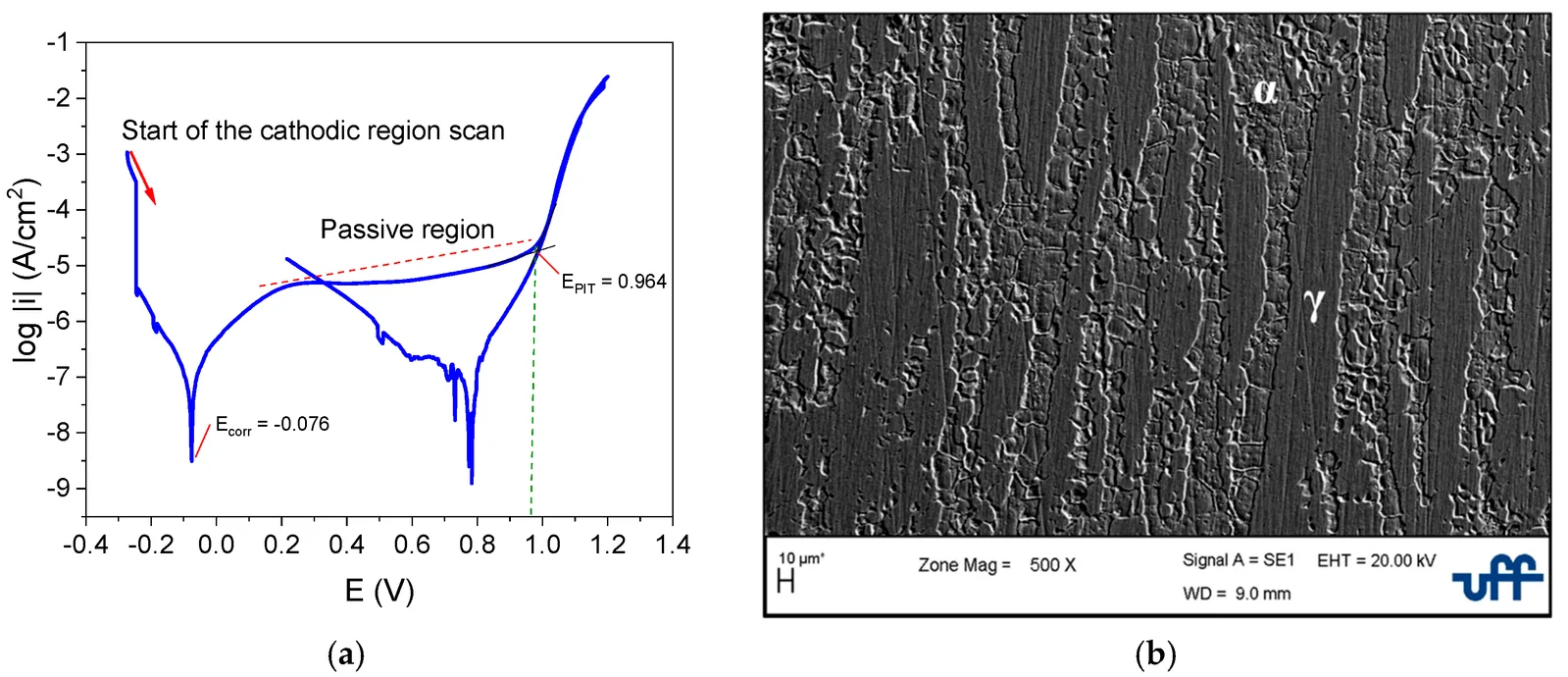
(**a**) Polarization curve of the as-received sample; (**b**) Micrograph after electrochemical testing showing the phases present: austenite (γ) and ferrite (α).

**Figure 5:**
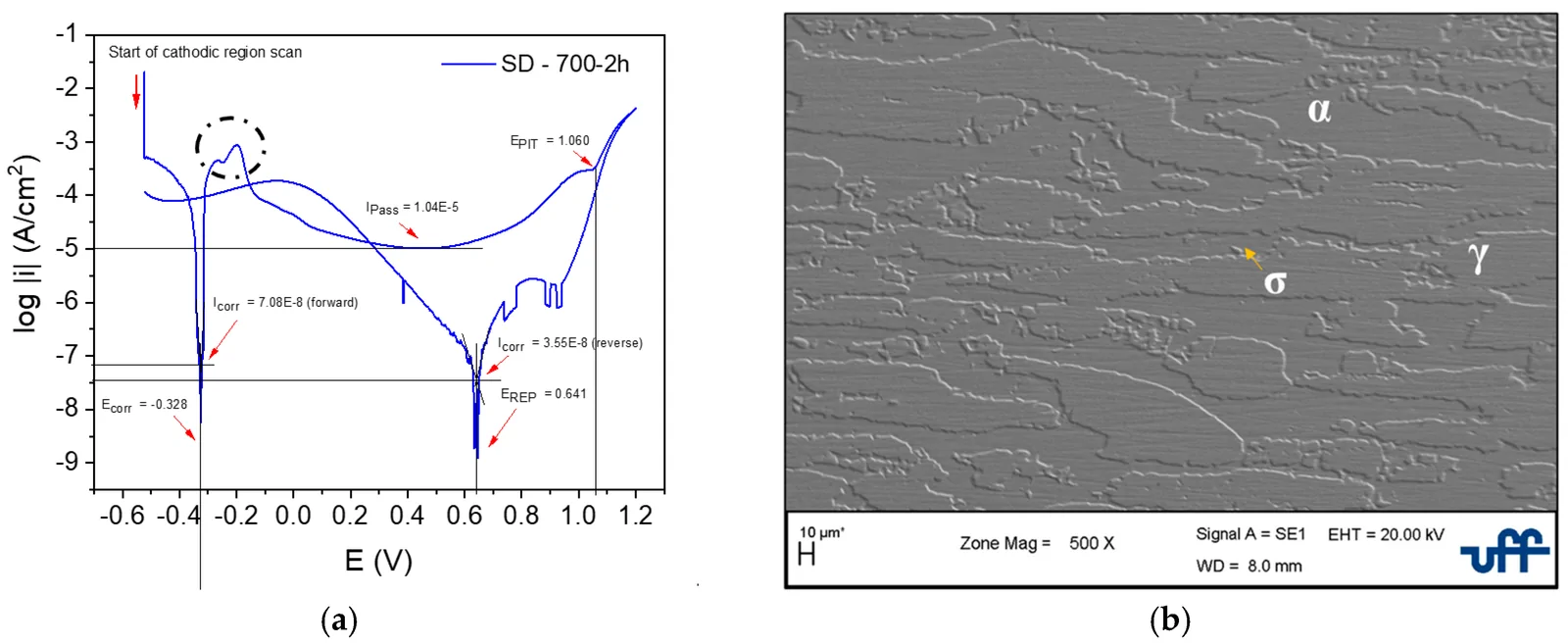
(**a**) Polarization curve of the Superduplex stainless steel sample heat-treated at 700 °C for 2 h; (**b**) Micrograph after electrochemical testing showing the phases present: austenite (γ), ferrite (α), and sigma (σ).

**Figure 6:**
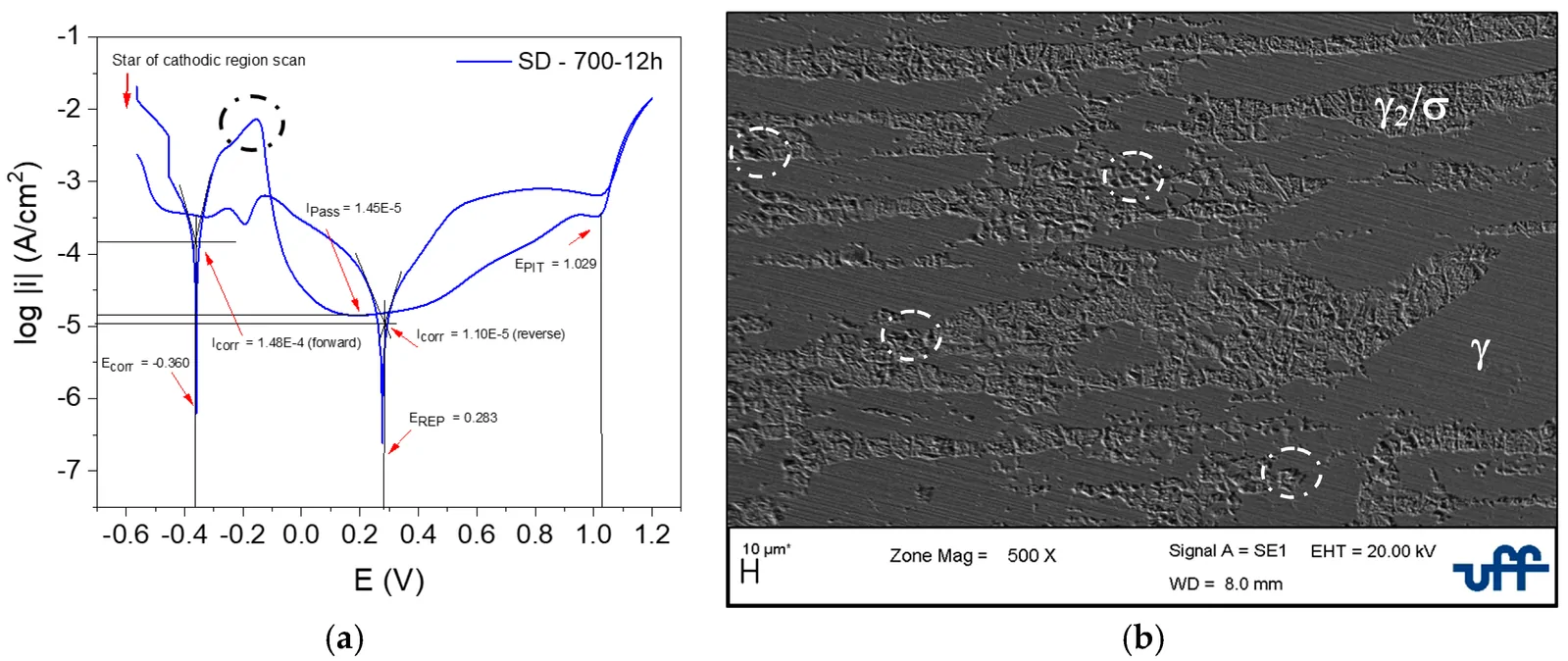
(**a**) Polarization curve of the Superduplex stainless steel sample heat-treated at 700 °C for 12 h; (**b**) Micrograph after electrochemical testing identifying the phases present: austenite (γ), secondary austenite (γ_2_), and sigma (σ) in lamellae.

**Figure 7:**
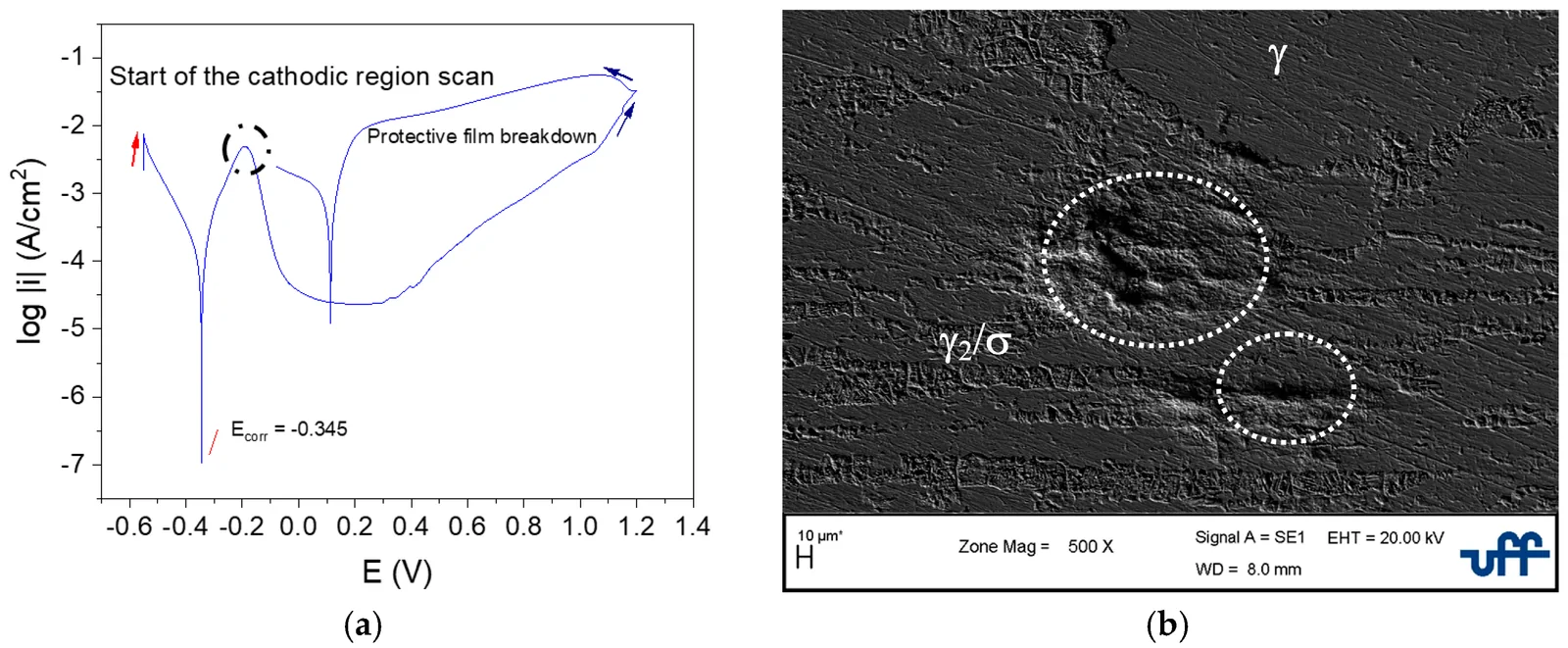
(**a**) Polarization curve of the Superduplex stainless steel sample heat-treated at 700 °C for 50 h; (**b**) Micrograph after electrochemical testing showing the phases present: austenite (γ), secondary austenite (γ_2_), and sigma (σ) in lamellae. The whites circles highlight the selected corroded area.

**Figure 8:**
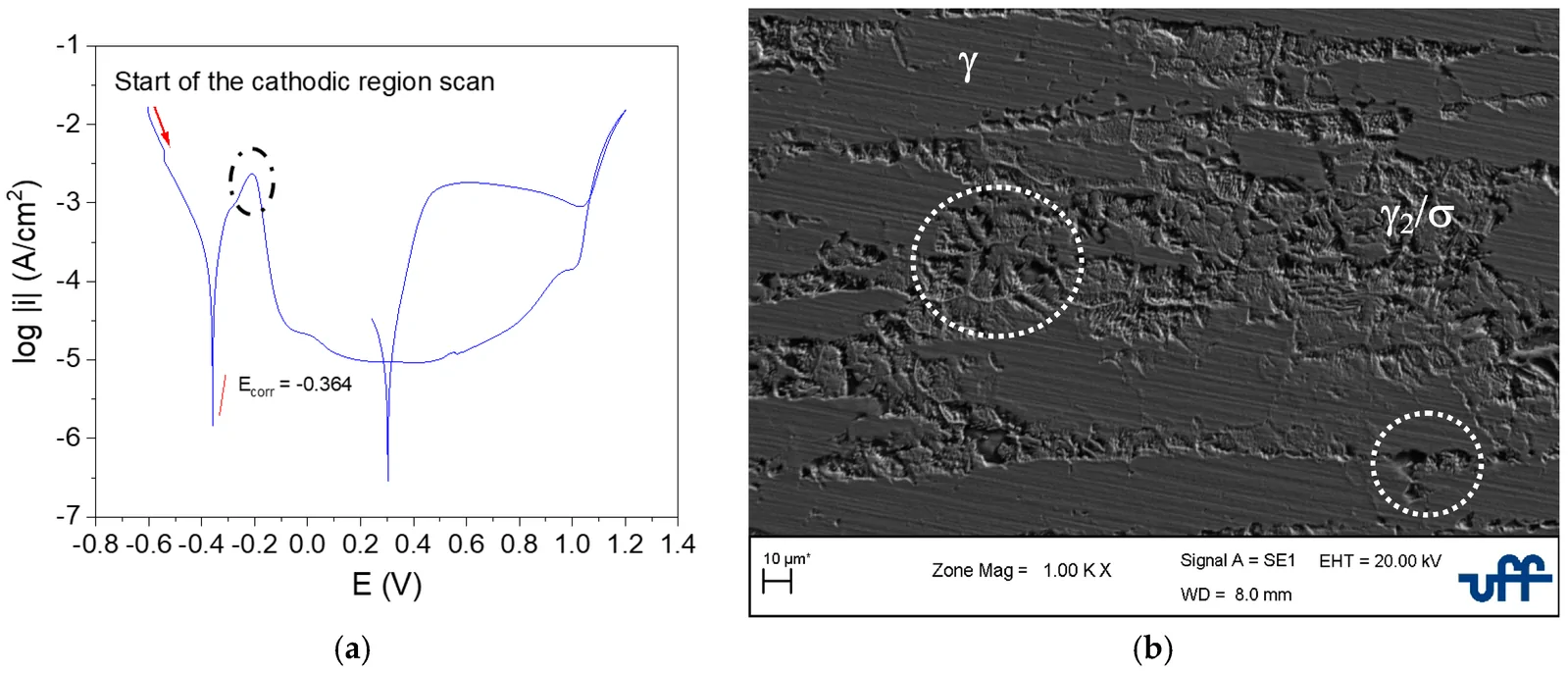
(**a**) Polarization curve of the Superduplex stainless steel sample heat-treated at 800 °C for 1 h; (**b**) Micrograph after electrochemical testing identifying the phases present: austenite (γ), secondary austenite (γ_2_), and sigma (σ) in lamellae. The whites circles highlight the selected corroded area.

**Figure 9:**
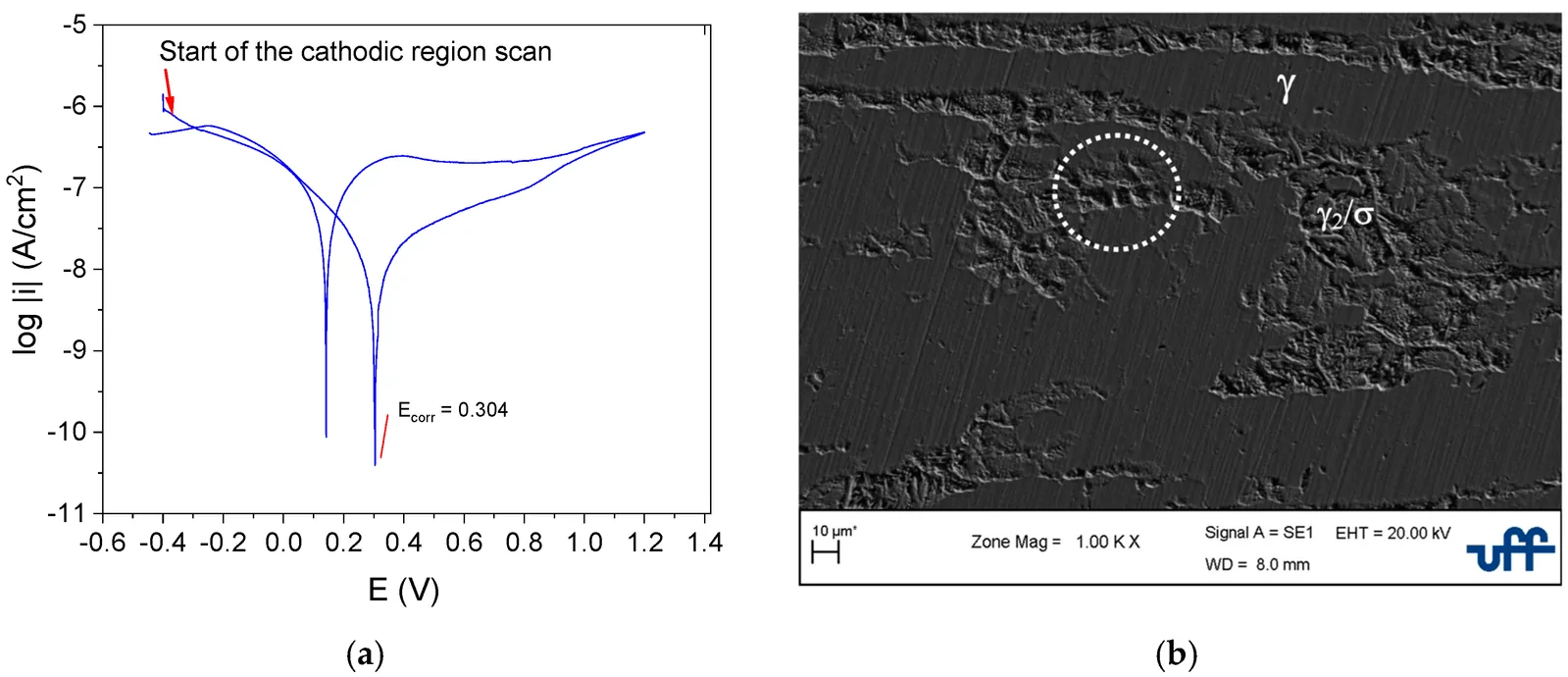
(**a**) Polarization curve of the Superduplex stainless steel sample heat-treated at 800 °C for 7.5 h; (**b**) Micrograph after electrochemical testing showing the phases present: austenite (γ), secondary austenite (γ_2_), and sigma (σ) in lamellae. The white circle highlights the selected corroded area.

**Figure 10:**
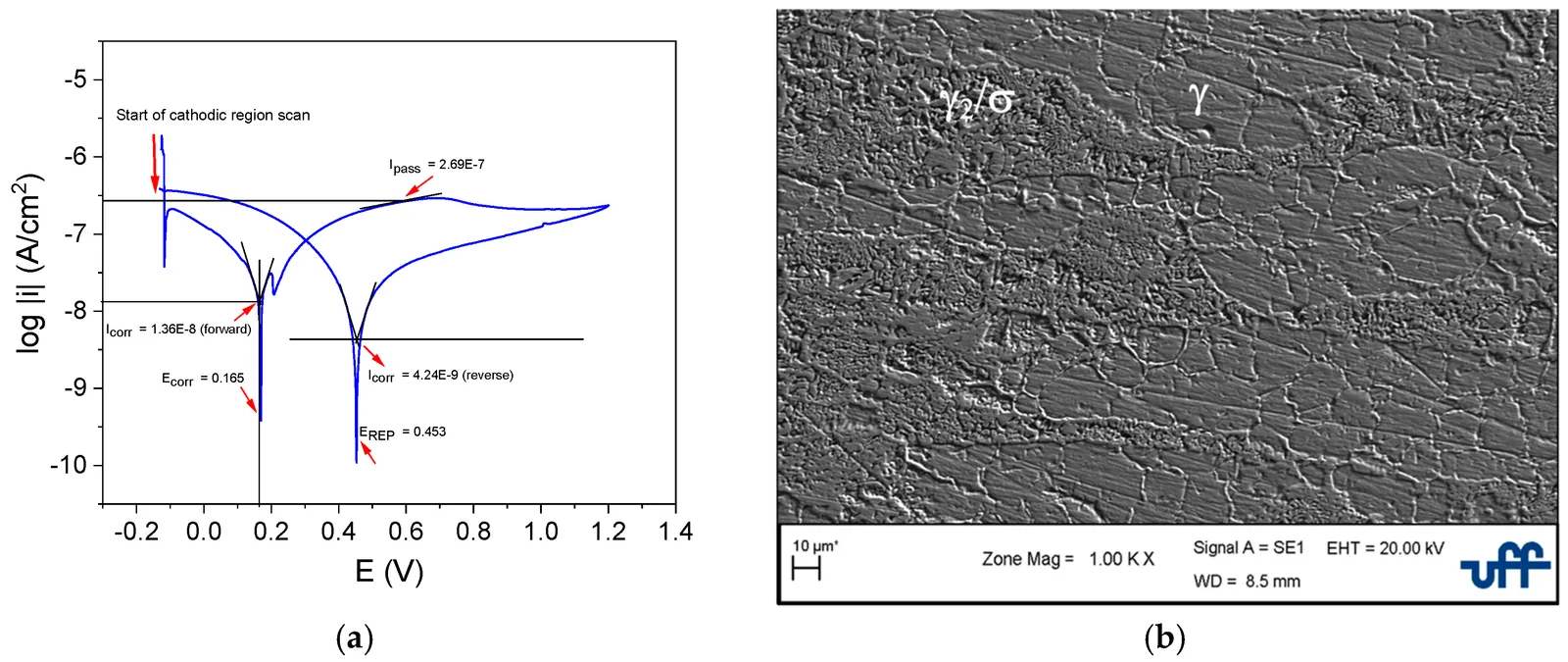
(**a**) Polarization curve of the Superduplex stainless steel sample heat-treated at 800 °C for 30 h; (**b**) Micrograph after electrochemical testing, identifying the phases present: austenite (γ), secondary austenite (γ_2_), and sigma (σ) in lamellae.

The polarization curves from the cyclic polarization tests are displayed below (Figure 4, Figure 5, Figure 6, Figure 7, Figure 8, Figure 9 and Figure 10). To support the electrochemical interpretation, each curve is accompanied by SEM micrographs of the corresponding regions tested.

Figure 4a shows the cyclic polarization curve of the as-received superduplex stainless steel. A broad passive region is observed, indicating the formation of a stable and protective passive film.

The electrochemical parameters extracted from the polarization curves, including the corrosion potential (Ecorr), corrosion current density (icorr), passive current density (ipass), pitting potential (Epit), and repassivation potential (Erep), are summarized in Table 3. For ease of comparison among the different aging conditions, all cyclic polarization curves are also presented together in Supplementary Figure S1.

Differences between Eocp, Table 2, and Ecorr, Table 3, may arise because Eocp is measured under open-circuit conditions. In contrast, Ecorr is determined from polarization curves in which the potential is continuously scanned, thereby perturbing the electrochemical system [6].

The negative hysteresis loop indicates that any localized breakdown of the passive film is followed by efficient repassivation, preventing the stable growth of pits. Furthermore, the large potential difference between Ecorr and Epit indicates that the as-received material has a high resistance to localized corrosion [6].

The SEM micrograph after testing (Figure 4b) reveals no evidence of pitting or selective attack, corroborating the electrochemical results and confirming the excellent corrosion resistance of the solution-annealed condition.

For the sample aged at 700 °C for 2 h (*Vv_σ_* = 0.02), an initial increase in current density is observed in Figure 5a, as highlighted by the dashed circle. Although the sigma phase fraction is relatively low, its precipitation may introduce microstructural heterogeneities and localized Cr- and Mo-depleted regions in the surrounding matrix, leading to a temporary increase in anodic dissolution. However, the current density rapidly decreases, indicating the formation of a stable passive film and the restoration of passivity.

The cyclic polarization curve exhibits a negative hysteresis loop, with the reverse scan remaining below the forward scan. This behavior indicates the absence of stable pit nucleation and growth during polarization. The lack of pitting susceptibility is further supported by the surface morphology in Figure 5b, which shows no evidence of localized corrosion.

For the sample aged at 700 °C for 12 h (*Vv_σ_* = 0.17), Figure 6a shows a more pronounced increase in current density than the sample aged for 2 h (Figure 5a), as indicated by a dashed circle. This behavior is likely associated with the higher volume fraction of sigma phase, which promotes greater microstructural heterogeneity and the formation of Cr- and Mo-depleted regions around the precipitates. Nevertheless, the current density subsequently decreases markedly, indicating the formation of a stable passive film and the restoration of passivity. Similar relationships between σ-phase precipitation, passive film characteristics, and corrosion resistance have also been reported in stainless steels, where the presence of σ phase significantly influences the stability and repair of the passive film [33,34,35].

Unlike the sample aged for 2 h, the cyclic polarization curve exhibits a positive hysteresis loop, suggesting that localized corrosion was initiated during the forward scan and continued to propagate during the reverse scan. This behavior indicates stable pit formation and reduced resistance to pitting corrosion. The presence of localized corrosion is confirmed by the surface morphology shown in Figure 6b, where pits are observed preferentially in regions containing secondary austenite lamellae associated with sigma phase precipitation. These microstructural regions are known to be depleted in chromium and molybdenum, making them preferential sites for pit nucleation. 

The sample aged at 700 °C for 50 h (Figure 7a) exhibits a polarization curve markedly different from those obtained under the previous aging conditions. In this case, a pronounced positive hysteresis loop is observed, similar to that found for the sample aged at 700 °C for 12 h (Figure 6a), indicating susceptibility to localized corrosion. Analysis of the current densities during the forward and reverse scans (Table 3) reveals that the reverse-scan current density remains higher than the forward-scan current density over a wide potential range, confirming the occurrence of stable pitting corrosion [6].

The electrochemical behavior of the sample aged at 700 °C for 50 h can be attributed to extensive microstructural changes induced by thermal exposure. Under these conditions, a significant volume fraction of sigma phase (*Vv_σ_* = 0.36) is formed, accompanied by the precipitation of secondary austenite (γ_2_) and the development of chromium- and molybdenum-depleted regions adjacent to the sigma particles. These microstructural features generate pronounced electrochemical heterogeneities, making the depleted regions and secondary austenite preferential sites for localized attack.

Figure 7a shows that during the initial stage of anodic polarization, preferential dissolution of these less corrosion-resistant regions may occur before a stable passive film forms, as highlighted by the dashed circle. As the potential increases, passivation occurs via the formation of a protective chromium-rich oxide layer. However, at higher potentials, passive film breakdown occurs, resulting in a sharp increase in current density associated with pit nucleation and growth.

The micrograph shown in Figure 7b reveals selective corrosion preferentially occurring in regions containing sigma phase and secondary austenite. The localized attack observed between the sigma lamellae is consistent with the electrochemical results. It supports the hypothesis that chromium- and molybdenum-depleted areas surrounding the sigma phase are the preferential sites for pit initiation. These findings highlight the importance of correlating electrochemical measurements with microstructural characterization to accurately identify the dominant corrosion mechanisms.

For the sample aged at 800 °C for 1 h (*Vv_σ_* = 0.23), an initial increase in current density is observed in Figure 8a, as highlighted by the dashed circle. This increase is more pronounced than that observed for the sample aged at 700 °C for 2 h (Figure 5a) and is attributable to the higher volume fraction of sigma phase formed during aging. The precipitation of sigma phase, together with the formation of chromium- and molybdenum-depleted regions and secondary austenite (γ_2_), promotes electrochemical heterogeneities that favor preferential anodic dissolution during the initial stages of polarization. However, the current density subsequently decreases, indicating the formation of a passive film and the establishment of a passive state.

The polarization curve (Figure 8a) exhibits a positive hysteresis loop, suggesting localized corrosion during anodic polarization. However, unlike the sample aged at 700 °C for 50 h (Figure 7a), the icorr measured during the reverse scan is not consistently higher than that observed during the forward scan. Therefore, the electrochemical response alone does not provide unequivocal evidence of stable pit growth. In this case, the microstructural observations become particularly important.

The surface morphology shown in Figure 8b reveals localized corrosion preferentially occurring between sigma phase and secondary austenite lamellae. These regions are known to be depleted in chromium and molybdenum due to sigma-phase precipitation, making them more susceptible to anodic dissolution. The selective attack observed in these areas confirms that the positive hysteresis is associated with localized corrosion processes and demonstrates the detrimental effect of the microstructural changes induced by aging at 800 °C. Similar observations were reported by Sampaio et al. [36], who used AFM after corrosion testing and showed that the sigma phase stands topographically higher than the surrounding corroded regions, due to Cr and Mo depletion in its vicinity.

The specimen, aged at 800 °C for 7.5 h and containing approximately 25% sigma phase, exhibited an electrochemical response distinct from those observed under the previous aging conditions. As shown in Figure 9a, the polarization curve does not display a clearly defined passive region or a well-defined pitting potential. Instead, a positive hysteresis loop is observed, with the current density during the reverse scan remaining higher than that measured during the forward scan over a broad potential range. This behavior indicates limited repassivation and suggests that localized corrosion processes continue to propagate after initiation.

The corresponding SEM micrograph (Figure 9b) reveals localized attack preferentially occurring at γ/σ and γ_2_/σ interfaces. The attacked regions are associated with chromium- and molybdenum-depleted zones that form during sigma-phase precipitation and secondary austenite formation. These microstructural heterogeneities promote preferential anodic dissolution and act as favorable sites for pit nucleation and growth.

Interestingly, despite having a sigma phase volume fraction (*Vv_σ_* ≈ 0.25) similar to that of the specimen aged at 800 °C for 1 h (*Vv_σ_* ≈ 0.23), the electrochemical response differs considerably. This observation suggests that corrosion resistance is not controlled solely by the amount of sigma phase but is also strongly influenced by the evolution of secondary austenite, elemental redistribution, and the resulting local chemical heterogeneities.

Furthermore, as shown in Figure 3, the specimen aged at 800 °C for 7.5 h initially exhibited a cyclic “corrode–does not corrode” behavior based on the open-circuit potential measurements. However, the cyclic polarization results together with the microstructural observations demonstrate that this condition is highly susceptible to localized corrosion.

For the sample treated at 800 °C for 30 h (*Vv_σ_* = 0.33), the polarization curve (Figure 10a) exhibits a stable passive region with a low passive current density and a negative hysteresis loop. No distinct pitting potential was observed within the investigated potential range, indicating that passive film breakdown was not sustained under the test conditions. Furthermore, the lower current density during the reverse scan suggests efficient repassivation of any metastable defects formed during anodic polarization.

The SEM micrograph after testing (Figure 10b) revealed no evidence of pitting or selective corrosion, corroborating the electrochemical results and indicating that the passive film remained stable throughout the experiment. Figure 10b shows a micrograph of the sample, where the phases are well preserved, and no corrosion is observed.

An interesting comparison can be made with the specimen aged at 700 °C for 50 h (*Vv_σ_* = 0.36), Figure 7a,b, which exhibited selective corrosion despite having a σ-phase fraction similar to that of the 800 °C/30 h condition. This result suggests that corrosion resistance is controlled not solely by the amount of σ phase but also by the local chemical redistribution associated with its precipitation.

At 800 °C, the higher chromium diffusivity promotes a more effective redistribution of chromium [19,37] around the σ phase during prolonged aging, reducing the severity of chromium-depleted regions. In contrast, the lower diffusivity at 700 °C limits rehomogenization, thereby preserving more pronounced chromium-depleted zones that serve as preferential sites for localized corrosion.

#### 3.2.3. Double-Loop Electrochemical Potentiodynamic Reactivation (DL-EPR) Tests

To complement the electrochemical characterization from cyclic polarization, the degree of sensitization (DOS) was evaluated using the Double Loop Electrochemical Potentiodynamic Reactivation (DL-EPR) technique. This method enables quantification of chromium depletion related to secondary-phase precipitation, particularly the σ phase, by comparing the activation (i_a_) and reactivation (i_r_) current densities [17,38]. DL-EPR tests were performed in quintuplicate, and the results showed good reproducibility, with variability in iₐ and iᵣ within ±15%. The DOS values reported in this study were calculated from the average activation and reactivation current densities obtained from the five independent measurements.

**Figure 11:**
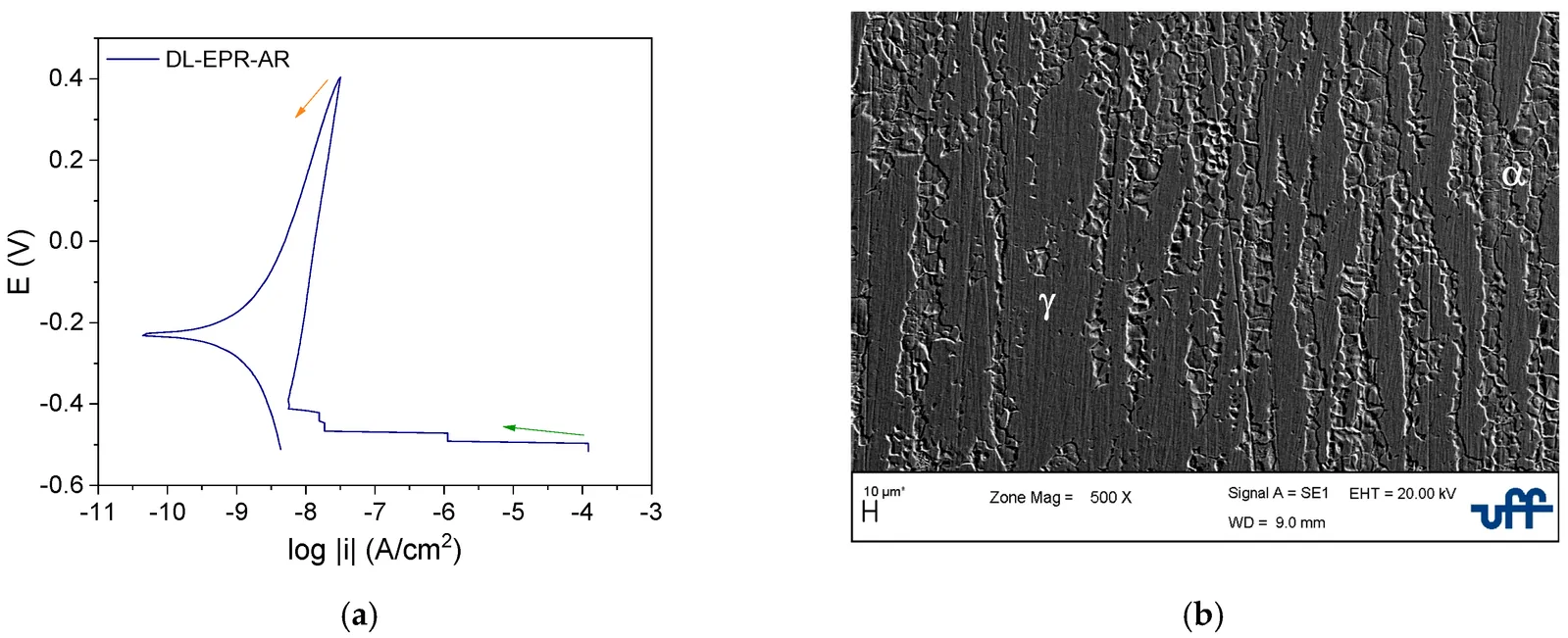
(**a**) DL-EPR graph for the as-received Superduplex stainless steel sample; the green arrow shows the start of the anodic scan, and the orange arrow indicates the cathodic scan. (**b**) Surface of the sample after electrochemical testing. α—ferrite and γ—austenite.

Figure 11a shows the typical DL-EPR curve for superduplex stainless steel in its as-received condition. No reactivation peak is observed, indicating the absence of intergranular corrosion and confirming that the material remains fully passivated with a stable protective film. The micrograph in Figure 11b shows no signs of selective attack at the ferrite/austenite interfaces. This is expected, since in the as-received state, no intermetallic phases are present; therefore, chromium depletion does not occur, allowing the passive layer to stay intact.

**Figure 12:**
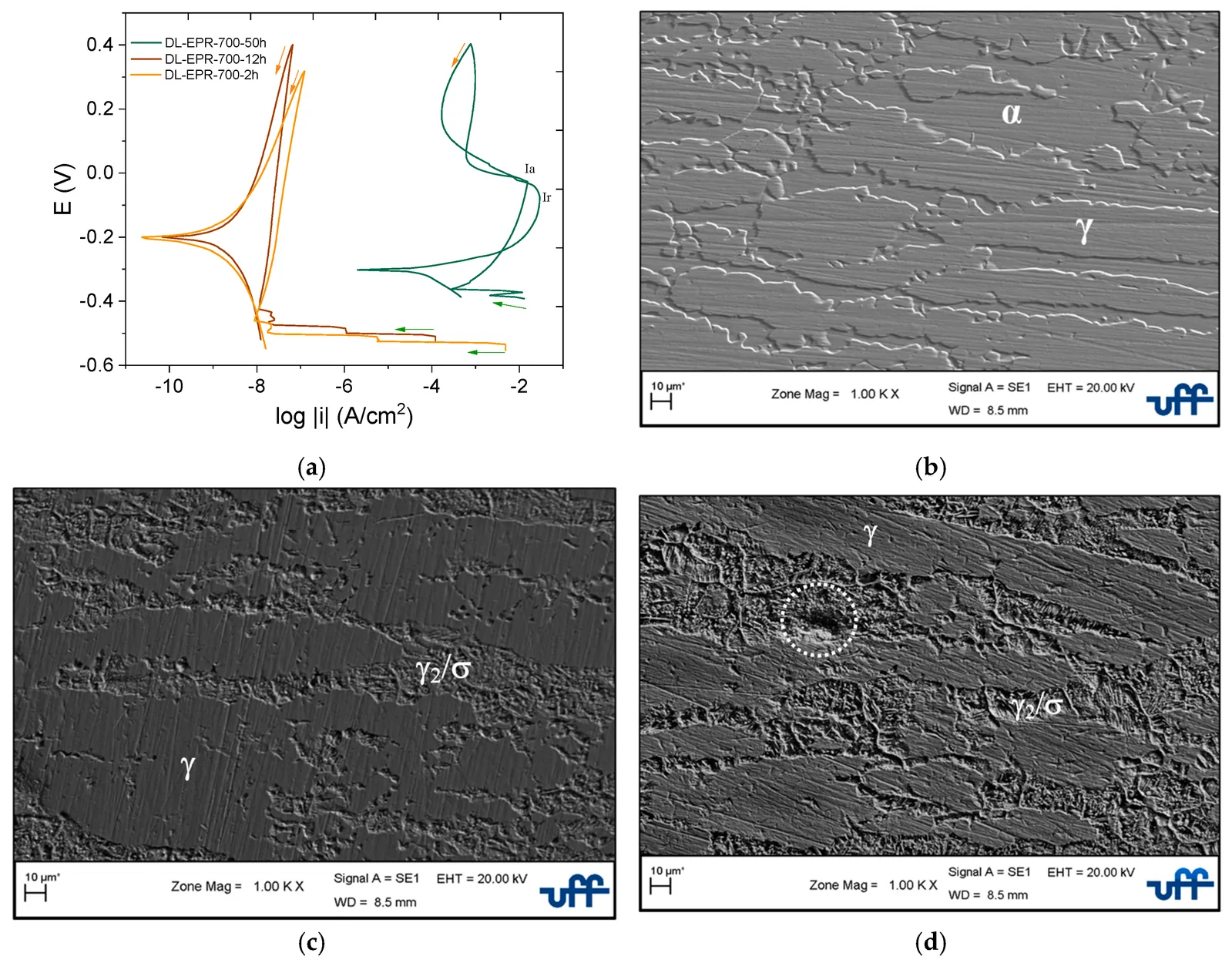
(**a**) DL-EPR graph for superduplex stainless steel samples heat-treated at 700 °C for 2 h, 12 h, and 50 h, with a green arrow indicating the start of the anodic scan and an orange arrow showing the reversal in the cathodic direction. (**b**) Surface of the sample treated at 700 °C after electrochemical testing: (**b**) 2 h, (**c**) 12 h, and (**d**) 50 h. Austenite (γ), secondary austenite (γ_2_), and sigma (σ) in lamellae. In (**d**), the white circle highlights the selected corroded area.

Analyzing the DL-EPR curves for the superduplex samples treated at 700 °C for 2 h, 12 h, and 50 h (Figure 12a–d), it is observed that no activation or reactivation peaks appear for the samples aged for 2 h and 12 h. Their curves display a profile similar to that of the as-received condition (Figure 11a), indicating the absence of chromium depletion severe enough to cause intergranular corrosion.

At 700 °C, the samples aged for 1 and 12 h (Figure 12a) remain passive and show no selective corrosion at the grain boundaries (Figure 12b,c). For the 1 h condition, the low sigma fraction (≈2%) is not enough to cause noticeable chromium depletion. For the 12 h condition, although the sigma fraction rises to about 17%, chromium depletion around σ would typically be expected; however, 700 °C is a relatively low temperature for sigma-phase precipitation kinetics [39,40]. At this temperature, chromium diffusion is restricted, preventing the formation of a depletion zone strong enough to cause a reactivation peak or intergranular attack.

For the sample treated at 700 °C for 50 h, activation and reactivation current density peaks are observed, as shown in Figure 12a. However, sensitization does not occur; instead, selective corrosion develops between the sigma-phase lamellae. In this condition (*Vv_σ_* = 0.36), the sigma phase forms as lamellae along with secondary austenite (Figure 1d and Figure 12d), which aligns with the microstructural morphologies reported for superduplex steels aged at low temperatures [9].

Although the DL-EPR methodology follows the same process as the studies mentioned earlier, the test can yield a false positive when localized corrosion occurs outside grain boundaries. In our case, corrosion initiates between sigma-phase lamellae (Figure 12d), and true intergranular sensitization is not observed because Cr depletion remains highly localized around sigma-phase lamellae without affecting ferrite–austenite grain boundaries. Therefore, post-test microstructural examination is essential for accurately interpreting DL-EPR results and for avoiding the misidentification of localized lamellar corrosion as sensitization.

The DL-EPR technique is fundamentally sensitive to the electrochemical reactivation of chromium- or molybdenum-depleted regions rather than to grain boundaries themselves. During the reverse scan, preferential dissolution occurs wherever local chromium depletion weakens the passive film, generating a reactivation current. Previous studies have shown that the reactivation peak corresponds to the dissolution of Cr- or Mo-depleted regions adjacent to σ-phase precipitates rather than exclusively to grain-boundary attack [41]. Recent studies have likewise demonstrated that σ-phase precipitation promotes local chemical heterogeneities that govern the sensitization behavior detected by DL-EPR and promote intergranular corrosion [42].

Consistent with these observations, the elevated DL-EPR response observed in the present study arises because the electrochemical signal originates from chromium-depleted zones adjacent to σ-phase lamellae rather than from ferrite–austenite grain boundaries. In lamellar σ structures, alternating regions of σ (rich in Cr and Mo) and secondary austenite (rich in Ni) generate localized chromium-depleted regions that act as preferential dissolution paths during the reactivation scan. Although these interfaces are intragranular, their electrochemical behavior mimics the classical activation–reactivation response observed in conventionally sensitized stainless steels. This interpretation is consistent with the findings of Hosseini et al. [22], who demonstrated that the morphology of the σ phase strongly influences the degradation mechanism and the location of localized corrosion. Consequently, the reactivation peak observed in Figure 12d reflects selective dissolution between σ-phase lamellae rather than true intergranular sensitization. Therefore, the high DOS value (188%) should be interpreted as a consequence of the morphology and connectivity of the lamellar σ network rather than as evidence of conventional intergranular corrosion. The sample surfaces after electrochemical testing are shown in Figure 12b–d.

**Figure 13:**
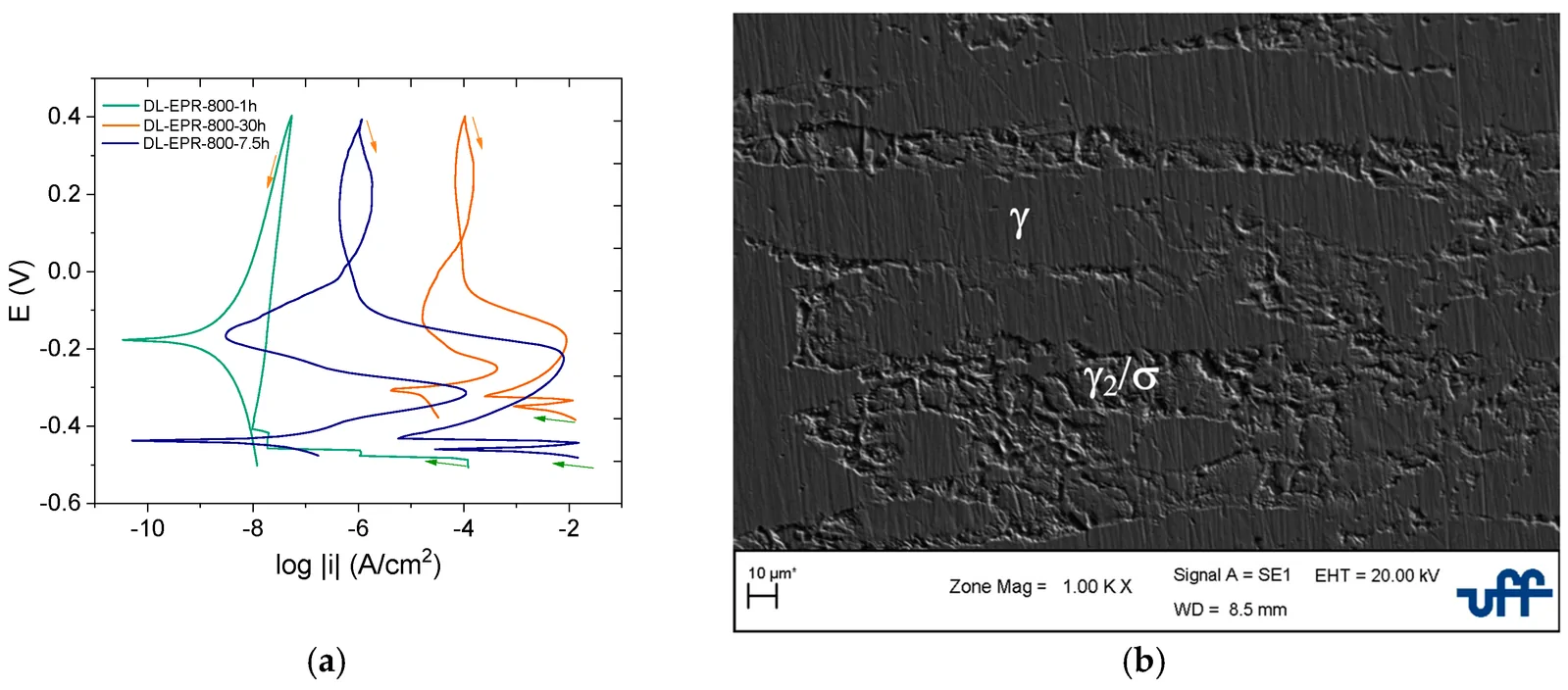

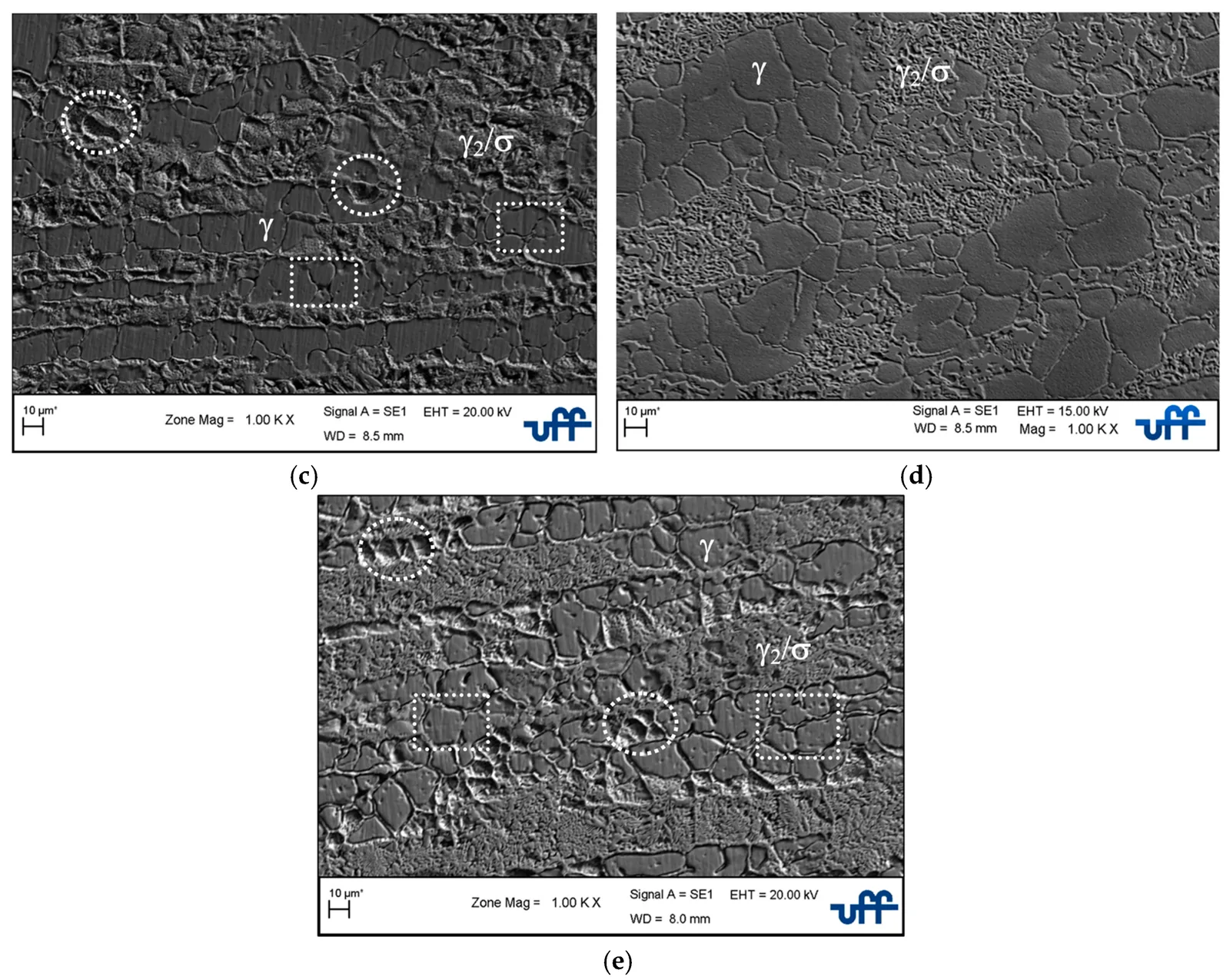
(**a**) DL-EPR curves for superduplex stainless steel samples heat-treated at 800 °C for 1.0 h, 7.5 h, and 30 h. The green arrow indicates the start of the anodic scan, and the orange arrow shows the reversal toward the cathodic direction. (**b**) Surface of the sample treated at 800 °C for 1.0 h after the electrochemical test. (**c**) Surface of the sample treated at 800 °C for 7.5 h after the electrochemical test. (**d**) Surface of the sample treated at 800 °C for 30 h before the electrochemical test. (**e**) Surface of the sample treated at 800 °C for 30 h after the electrochemical test. In (**c**,**e**), the white circles highlight the corroded area in sigma lamellae. The white rectangles show corrosion at grain boundaries.

Figure 13a shows the DL-EPR curves for superduplex stainless steel samples treated at 800 °C for 1.0, 7.5, and 30 h. Clear differences in their electrochemical behavior are apparent.

For the 1.0 h condition, the DL-EPR curve shows that no sensitization took place, even though the sigma-phase volume fraction is relatively high (*Vv_σ_* = 0.23). This suggests that, at 800 °C and for short exposure times, chromium depletion around sigma-phase particles stays limited. Although sigma is already present in a significant volume, the diffusion distances needed to cause critical Cr depletion at ferrite/austenite boundaries have not yet been reached. As a result, no reactivation peak is observed, indicating the absence of intergranular corrosion.

For the 7.5 h and 30 h conditions, the DL-EPR curves (Figure 13a) display prominent reactivation peaks, with intensities increasing as the heat-treatment time extends. The sigma-phase fractions in these samples (*Vv_σ_* = 0.25 and *Vv_σ_* = 0.33, respectively) are enough to create well-developed Cr-depleted regions in the surrounding ferrite, encouraging reactivation during the DL-EPR test. As a result, the degree of sensitization steadily increases with increasing exposure time at 800 °C. DOS = 11.9% and 27.9% for samples treated at 800 °C for 7.5 h and 30 h, respectively.

These results indicate that at 800 °C, sensitization depends not only on the sigma fraction but also on the duration available for Cr redistribution and depletion around σ particles. Short durations result in a high σ-phase fraction but insufficient Cr depletion (1.0 h), whereas longer durations yield both higher σ-phase fractions and more pronounced Cr-depleted zones, leading to measurable reactivation (7.5 h and 30 h).

Figure 13b,c display the microstructure of the samples treated for 1.0 h and 7.5 h after the DL-EPR test. As observed, the sample treated for 1.0 h shows no signs of intergranular corrosion, indicating the absence of chromium-depleted grain boundaries.

For the sample treated for 7.5 h (Figure 13c), selective attack is still observed within the σ-phase lamellae, but a continuous attack develops along grain boundaries containing σ-phase, indicating sensitization.

Figure 13d,e present representative SEM micrographs of the same exposed region of the sample treated for 30 h before and after the DL-EPR test. Although the images do not correspond to exactly the same microscopic field, they illustrate the characteristic microstructural condition before testing and the corrosion morphology developed after the electrochemical test. As shown in Figure 13e, corrosion occurs both between the σ-phase lamellae—due to local chromium-depleted regions at σ/secondary austenite—and along grain boundaries containing σ-phase, where continuous dissolution indicates advanced degradation sensitization.

The results of DL-EPR tests generally indicate that the as-received superduplex steel did not exhibit intergranular corrosion; the material remained passive throughout the test (Figure 11a,b). At 700 °C, the samples treated for 2 h and 12 h also showed passivation, with no activation or reactivation peaks, and no corrosion was observed at ferrite/austenite grain boundaries (Figure 12a–c).

During the 50 h treatment at 700 °C, activation and reactivation peaks appear (Figure 12a), indicating a very high calculated degree of sensitization (DOS = 188%). However, the micrograph (Figure 12d) shows that the attack occurs between σ-phase lamellae rather than along grain boundaries. This confirms that the DL-EPR signal is a “false positive,” highlighting the importance of always examining the microstructure after electrochemical testing.

At 800 °C, the 1 h sample exhibits no activation–reactivation peaks, indicating no sensitization (Figure 13a,b). For treatment times of 7.5 h and 30 h, clear activation and reactivation peaks are seen (Figure 13a), confirming sensitization. The post-test surfaces (Figure 13c–e) show corrosion at grain boundaries containing σ-phase, along with selective dissolution between the σ lamellae.

Overall, the combined DL-EPR and microstructural analysis shows that the σ-phase lamellar morphology causes selective corrosion, whereas the σ-phase at grain boundaries leads to significant sensitization.

#### 3.2.4. Cr Distributions

To better understand the relationships among σ-phase morphology, σ-phase fraction, and the resulting corrosion mode, a statistical analysis of chromium distribution was performed after the DL-EPR test for the as-received condition (AR) and for the three heat-treatment conditions that exhibited corrosion during the DL-EPR test. The AR sample was used as the reference condition, while the 700—50 h, 800—7.5 h, and 800—30 h samples were selected because they showed corrosion associated with chromium depletion after electrochemical testing. For each condition, 461 measurement points were collected: the as-received sample (AR, without σ), the 700—50 h sample (*Vv_σ_* = 0.36), the 800—7.5 h sample (*Vv_σ_* = 0.25), and the 800—30 h sample (*Vv_σ_* = 0.33).

The AR sample showed no corrosion (Figure 11b). The sample exposed to 700 °C for 50 h showed selective attack on the σ-phase lamellae (Figure 12d). In contrast, the samples treated at 800 °C for 7.5 h and 30 h exhibited a mixed corrosion mode: selective corrosion between σ-phase lamellae along with sensitization at grain boundaries containing σ-phase (Figure 13c,e).

**Figure 14:**
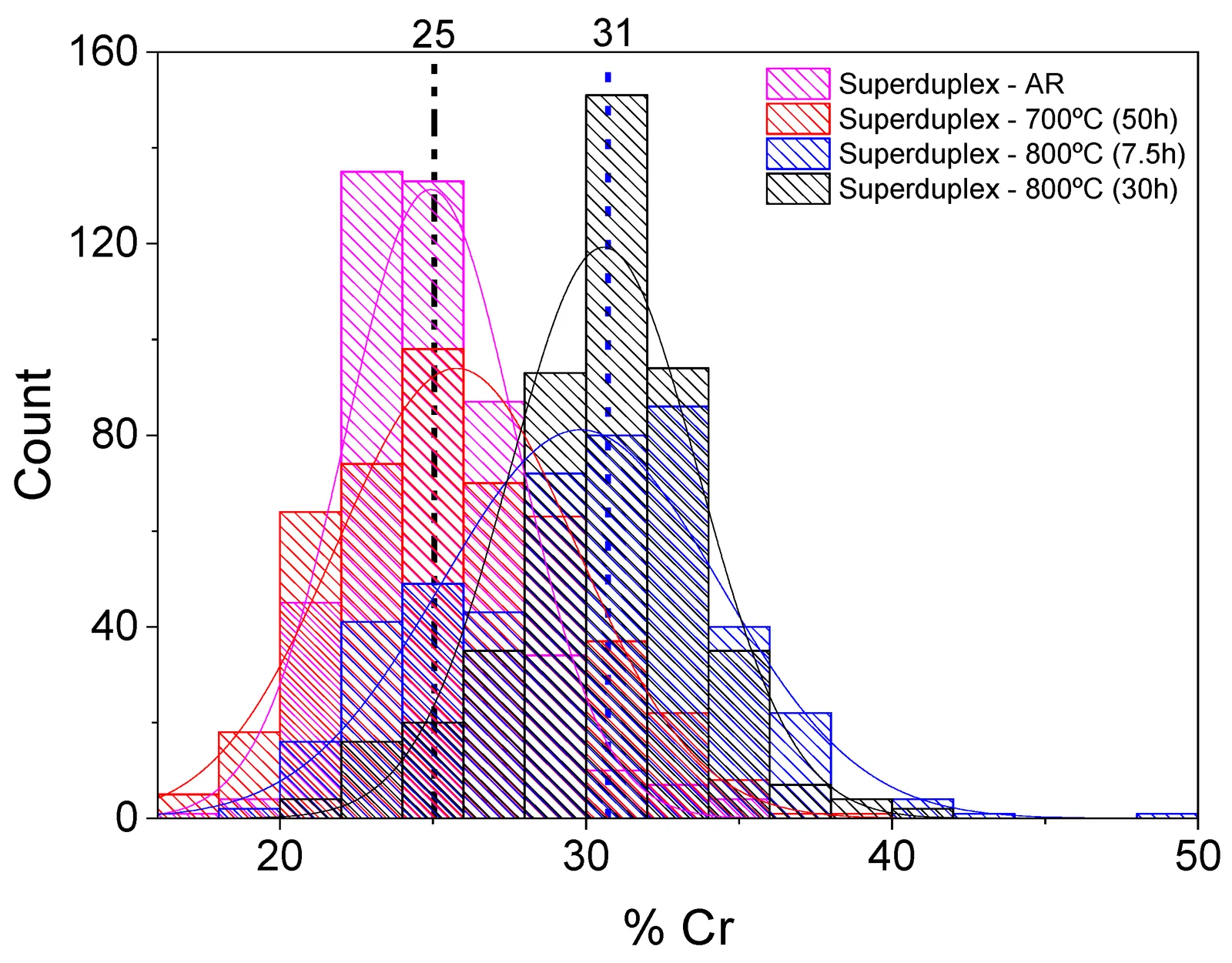
Cr distribution (%mass) after DL-EPR tests.

Figure 14 presents the statistical distribution of chromium for all four conditions after DL-EPR testing, revealing how Cr-depleted regions evolve as a function of both σ-phase volume fraction and morphology. For each condition, the chromium distributions shown in Figure 14 were obtained from 461 EDS point measurements automatically generated along line scans positioned across the corrosion-affected regions after the DL-EPR test.

The AR sample distribution, shown in pink, illustrates the distribution of chromium content. Its average value is approximately 25 wt.% Cr, consistent with the nominal chemical composition of the steel (Table 1). Using this bin (the ~25 wt.% Cr group) as a reference, a noticeable decrease in the number of measurements at 25 wt% Cr is observed in the other conditions. This reduction indicates the formation of Cr-depleted regions associated with σ-phase precipitation and the resulting microstructural changes.

For the 25 wt.% Cr class, the AR sample records 133 counts, while the samples treated at 700—50 h, 800—7.5 h, and 800—30 h show 98, 49, and 20 counts, respectively—this consistent decline in measurements around 25 wt.% Cr is accompanied by a notable increase in the overall width of the chromium distributions for the heat-treated samples, indicating the formation of Cr-depleted and Cr-enriched regions related to σ-phase precipitation.

Examining the micrographs in Figure 11b, Figure 12d and Figure 13c–e, and the distributions in Figure 14, it can be concluded that the roughly 25% decrease in Cr levels and the variation in distributions are linked to selective corrosion occurring between the sigma phase lamellae. This corrosion mode was demonstrated in Figure 12d and Figure 13c–e.

It is noteworthy that the average Cr content of the AR sample and that of the specimen aged at 700 °C for 50 h remain essentially similar. However, the decrease in the number of measurement points showing 25% Cr, along with a slight increase in variation, seems sufficient to promote selective corrosion along the sigma lamellae (Figure 12d). Despite this, the small difference in the average Cr concentration indicates that the driving force for sensitization under these conditions remains too weak.

When analyzing the samples aged at 800 °C, it becomes clear that, in addition to a significant reduction in Cr measurements of about 25%, the mean Cr values in these distributions increase to about 31%, compared to the AR and 700—50 h samples. The temperature effect plays a key role in this. Even the specimen treated for a shorter time at 800 °C (7.5 h) shows a higher average Cr content than the sample aged at 700 °C for 50 h.

In other words, higher temperature promotes Cr diffusion, leading to the formation of a sigma phase enriched in Cr. Similar findings were reported by Nagae et al. [43], who observed that the sigma phase in DSS can contain Cr levels that exceed those in the bulk alloy.

Furthermore, in addition to the selective corrosion between sigma lamellae observed in the 700—50 h sample, the specimens aged at 800 °C also showed clear signs of sensitization, as shown in Figure 13c,e. In these samples, the driving force for sensitization at the grain boundaries is quite clear. The higher Cr enrichment in the sigma phase at 800 °C results in a more pronounced Cr distribution gradient, promoting corrosion along the grain boundaries, where the sigma phase is mainly located.

## 4. Conclusions

This study emphasizes the importance of sigma-phase morphology and chromium distribution in affecting the corrosion behavior of superduplex stainless steel. Through microstructural analysis, electrochemical testing, and DL-EPR measurements, clear connections were established between the type, location, and amount of σ-phase precipitates and the observed corrosion mechanisms. The main conclusions from this work are summarized below.

- The morphology and distribution of the σ phase significantly affect the corrosion behavior of superduplex stainless steel.
- The lamellar (or coral-like) σ phase, formed through eutectoid decomposition at 700–800 °C, encourages selective corrosion between σ lamellae due to chromium-depleted regions surrounding the precipitates.
- The grain boundary σ phase, precipitated along α/γ interfaces, mainly causes sensitization and localized intergranular corrosion.
- For polarization tests, the sample aged at 700 °C for 50 h (*Vv_σ_* ≈ 0.36) showed selective corrosion between σ lamellae due to Cr depletion and limited Cr redistribution at this temperature. The sample aged at 800 °C for 30 h (*Vv_σ_* ≈ 0.33) did not exhibit pitting or selective corrosion, indicating that higher-temperature exposure improved Cr homogenization despite a high σ fraction.
- In summary, the statistical chromium analysis combined with the DL-EPR results demonstrates that both σ-phase morphology and chromium partitioning govern the corrosion mode. A moderate decrease in Cr around the σ lamellae is sufficient to promote selective attack, even when the mean Cr content remains unchanged (700—50 h). At higher temperatures (800 °C), enhanced Cr diffusion results in greater Cr enrichment in σ and more severe Cr depletion in the surrounding matrix, thereby intensifying the Cr gradient. This condition promotes not only lamellar-selective corrosion but also clear sensitization at σ-decorated grain boundaries.

## Figures and Tables

**Table 1:** Chemical Composition of UNS S32750 Superduplex Stainless Steel (wt. (%)).

C	Si	Mn	P	S	Cr	Ni	Mo	N	Cu
0.020	0.328	0.85	0.027	0.0009	24.89	6.82	3.72	0.278	0.156

**Table 2:** Open-circuit potential (OCP) test results.

Sample	Time	$E_{OCP}$ (V)
AR	-	−0.02 ± 0.01
700 °C	2 h	−0.15 ± 0.01
	12 h	−0.20 ± 0.01
	50 h	−0.38 ± 0.01
800 °C	1 h	−0.41 ± 0.02
	7.5 h	−0.55 ± 0.02
	30 h	0.03 ± 0.01

**Table 3:** Electrochemical parameters obtained from cyclic polarization curves for all tested superduplex stainless steel conditions. The values represent the mean of five independent measurements. The experimental variability of the electrochemical parameters was approximately ±10%.

Sample	Time	$i_{PASS}$ (A/cm^2^)	$i_{CORR}$ (A/cm^2^) (Forward)	$i_{CORR}$ (A/cm^2^) (Reverse)	$E_{CORR}$ (V)	$E_{PIT}$ (V)	$E_{REP}$ (V)	Hysteresis
AR	-	4.82 × 10^−6^	1.08 × 10^−7^	1.07 × 10^−8^	−0.076	0.964	0.783	negative
700 °C	2 h	1.04 × 10^−5^	7.08 × 10^−8^	3.55 × 10^−8^	−0.328	1.060	0.641	negative
	12 h	1.45 × 10^−5^	1.48 × 10^−4^	1.10 × 10^−5^	−0.360	1.029	0.283	positive
	50 h	2.34 × 10^−4^	1.48 × 10^−5^	8.91 × 10^−5^	−0.345	1.055	0.113	positive
800 °C	1 h	9.55 × 10^−6^	3.24 × 10^−5^	2.34 × 10^−6^	−0.364	1.028	0.304	positive
	7.5 h	-	5.75 × 10^−10^	6.03 × 10^−9^	0.304	-	0.142	positive
	30 h	2.69 × 10^−7^	1.36 × 10^−8^	4.24 × 10^−9^	0.165	-	0.453	negative

## Data Availability

The original contributions presented in this study are included in the article/Supplementary Material. Further inquiries can be directed to the corresponding author.

## Supplementary Materials

The following supporting information can be downloaded at: https://www.mdpi.com/article/10.3390/ma19163413/s1, Figure S1: Comparison of the cyclic polarization curves for all investigated conditions. This figure complements the individual polarization curves presented in Figure 4, Figure 5, Figure 6, Figure 7, Figure 8, Figure 9 and Figure 10, allowing direct comparison of the electrochemical behavior under all aging conditions. Curves with negative hysteresis are plotted using solid lines, whereas curves with positive hysteresis are plotted using dashed lines.

## References

[1] da Fonseca G.S., Mendes P.S.N., Silva A.C.M. (2019). Sigma phase: Nucleation and growth. Metals.

[2] Silva L.G.L., Naeem M., Costa T.H.C., Libório M.S., Bandeira R.M., Ferreira N.S., Rossino L.S., Júnior C.A., Queiroz J.C., Neto J.F. (2023). Wear and Corrosion of UNS S32750 Steel Subjected to Nitriding and Cathodic Cage Deposition. J. Mater. Eng. Perform..

[3] Acuna A., Riffel K.C., Ramirez A. (2024). A Comparison of Sigma Phase Formation in Solubilized Hyper Duplex Stainless Steel and Super Duplex Stainless Steel Filler Metals. Metall. Mater. Trans. A Phys. Metall. Mater. Sci..

[4] Oñate A., Toledo E., Ramirez J., Alvarado M.I., Jaramillo A., Sanhueza J.P., Medina C., Melendrez M.F., Rojas D. (2023). Production of Nb-doped super duplex stainless steel based on recycled material: A study of the microstructural characterization, corrosion, and mechanical behavior. Mater. Chem. Phys..

[5] Özbay Kısasöz B. (2025). Influence of initial grain size on the formation of sigma and its effect on corrosion and wear properties of 2205 duplex stainless steel. Mater. Chem. Phys..

[6] Sedriks A.J. (1996). Corrosion of Stainless Steels.

[7] Pratama A., Nurhaliza S., Santoso B., Dew R. (2025). Localized studies on pitting and crevice corrosion in marine-grade stainless steels: Experimental insights from Indonesia’s coastal industry. Int. J. Mater. Sci..

[8] Gudić S., Ćorić M., Vrsalović L., Nagode A., Krolo J., Jakić J. (2026). Effect of Natural Seawater Salinity on Stainless Steel Corrosion: Enhanced Resistance in Seawater Bittern. Appl. Sci..

[9] Pohl M., Storz O., Glogowski T. (2007). Effect of intermetallic precipitations on the properties of duplex stainless steel. Mater. Charact..

[10] Schuster R., Keplinger A., Jacob A., Kreyca J., Solyom L., Maawad E., Povoden-Karadeniz E. (2023). In-situ XRD investigation of σ phase precipitation kinetics during isothermal holding in a hyper duplex stainless steel. Mater. Charact..

[11] Gennari C., Pezzato L., Piva E., Gobbo R., Calliari I. (2018). Influence of small amount and different morphology of secondary phases on impact toughness of UNS S32205 Duplex Stainless Steel. Mater. Sci. Eng. A.

[12] Kobayashi D.Y., Wolynec S. (1999). Evaluation of the low corrosion resistant phase formed during the sigma phase precipitation in duplex stainless steels. Mater. Res..

[13] Dainezi I., Borges S.H., Mariano N.A. (2023). Effect of Precipitation of Alpha Line and Sigma Phases on the Microstructure and Corrosion Resistance of the Duplex Stainless Steel SAF 2205. Mater. Res..

[14] Li H., Liu W., Chen L., Sun Y., Zhang B., Zhou J., Wang F. (2024). Investigation of sigma phase generated by simulated welding heat treatment on corrosion behavior of 2205 hydraulic control pipeline steel. Int. J. Press. Vessels Pip..

[15] Zuo Y., Pan S., He B., Liang Z. (2024). Sigma phase embrittlement in a tempered Fe45Cr25Ni10Mn15Al4C1 medium-entropy alloy. Mater. Sci. Technol..

[16] Nilsson J.O. (1992). Super duplex stainless steels. Mater. Sci. Technol..

[17] Hong J., Han D., Tan H., Li J., Jiang Y. (2013). Evaluation of aged duplex stainless steel UNS S32750 susceptibility to intergranular corrosion by optimized double loop electrochemical potentiokinetic reactivation method. Corros. Sci..

[18] Quadros P.V.C.A., Ribeiro J.J.K., Schibicheski Kurelo B.C.E., Palma Calabokis O., Nuñez de la Rosa Y.E., Turin A.R., Borges P.C. (2024). Influence of Heat Treatment Temperature on Microstructure, Hardness and Sensitization of UNS S32205 Duplex Stainless Steel. Materials.

[19] de Sampaio M.T., Furtado A.B., Ignácio M.D.C., Tavares S.S.M., Pardal J.M., Pimenta A.R., Ponzio E.A. (2023). DL-EPR vs. LSV-KOH: From simple detection of deleterious phases to analysis of morphology and high accuracy on determination of sigma phase in UNS S32750 stainless steel. Corros. Eng. Sci. Technol..

[20] Pitacco E., Bertolini R., Ghinatti E., Pezzato L., Gennari C., Calliari I., Pigato M. (2024). Effect of open die forging and cooling rate on the precipitation of secondary phases and corrosion properties of a cast UNS S32760 super duplex stainless steel. J. Mater. Res. Technol..

[21] Breda M., Pezzato L., Pizzo M., Calliari I. (2014). Effect of cold rolling on pitting resistance in duplex stainless steels. La Metall. Ital..

[22] Hosseini V.A., Karlsson L., Wessman S., Fuertes N. (2018). Effect of sigma phase morphology on the degradation of properties in a super duplex stainless steel. Materials.

[23] Liu Z., Xie Y., Chu X., Zhang L., Zhao W., Zhao C., He H. (2023). Evolution of the sigma phase and its effect on the corrosion resistance of ASTM A890 3A duplex stainless steel. Mater. Today Commun..

[24] de Sampaio M.T., Furtado A.B., Ignacio M.D.C., Tavares S.S.M., Pardal J.M., Pimenta A.R., Velasco J.A., Ponzio E.A. (2023). Electrochemical characterization of sigma phase in superduplex stainless steels: LSV-KOH as a promising methodology over DL-EPR. J. Mater. Res. Technol..

[25] Da Fonseca G.S., de Oliveira P.M., Diniz M.G., Bubnoff D.V., de Castro J.A. (2017). Sigma Phase in Superduplex Stainless Steel: Formation, Kinetics and Microstructural Path. Mater. Res..

[26] Russ J.C., Dehoff R. (2000). Practical Stereology.

[27] Hwang H., Lee G., Jeon S., Park Y. (2009). Selective dissolution characteristics of 26cr-7ni-2.5mo-3w duplex stainless steel in H_2_SO_4_/HCI mixed solution. Mater. Trans..

[28] Bai G., Lu S., Li D., Li Y. (2015). Intergranular corrosion behavior associated with delta-ferrite transformation of Ti-modified Super304H austenitic stainless steel. Corros. Sci..

[29] Mészáros I., Bögre B., Szabó P.J. (2022). Magnetic and Thermoelectric Detection of Sigma Phase in 2507 Duplex Stainless Steel. Crystals.

[30] Tait W.S. (1994). An Introduction to Electrochemical Corrosion Testing for Practicing Engineers and Scientists.

[31] Berthomé G., Malki B., Baroux B. (2006). Pitting transients analysis of stainless steels at the open circuit potential. Corros. Sci..

[32] Shin B.H., Kim S., Park J., Ok J.W., Kim D., Yoon J.H. (2024). Study of Precipitated Secondary Phase at 700 °C on the Electrochemical Properties of Super Duplex Stainless Steel AISI2507: Advanced High-Temperature Safety of a Lithium-Ion Battery Case. Materials.

[33] Wang T.H., Wang J., Bai J.G., Wang S.J., Chen C., Han P.D. (2022). Effect of boron on dissolution and repairing behavior of passive film on S31254 super-austenitic stainless steel immersed in H_2_SO_4_ solution. J. Iron Steel Res. Int..

[34] Toor I.U. (2026). Advances in Understanding of Secondary Phases and Their Corrosion Implications in Stainless Steel Alloys—A Review. Corros. Mater. Degrad..

[35] Radojković B., Jegdić B., Pejić J., Eraković Pantović S., Marunkić D., Simović A., Milošević M., Alić B. (2025). Passive film properties and corrosion resistance of AISI 304L stainless steel. Corros. Eng. Sci. Technol..

[36] de Sampaio M.T.G., Furtado A.B., Ignácio M.D.C., Tavares S.S.M., Pardal J.M., Pimenta A.R., Cavalcanti E.H., Ponzio E.A. (2025). Quantification of Deleterious Phase Precipitation in a Hyper Duplex Stainless Steel Aged at 700–950 °C Using Optimized Linear Sweep Voltammetry: Effect of KOH Concentration. J. Mater. Eng. Perform..

[37] Silva R., Kugelmeier C.L., Martins Junior C.B., Oliveira P.H.F., Magalhães D.C.C., Plaine A.H., Magnabosco R., Rovere C.A. (2024). Mechanisms of intergranular corrosion and self-healing in high temperature aged lean duplex stainless steel 2404. npj Mater. Degrad..

[38] Ouyang M., Pan J., Wang Z., Ye X., Li J., Liu H.A., Xiao X. (2024). Influence of precipitation on the intergranular corrosion sensitivity of aged Alloy 31 using a modified electrochemical potentiokinetic reactivation method. Corros. Sci..

[39] Magnabosco R. (2009). Kinetics of Sigma Phase Formation In a Duplex Stainless Steel. Mater. Res..

[40] Acuna A., Ramirez A.J. (2023). Sigma phase formation kinetics in hyper duplex stainless steel welding filler metal. Mater. Charact..

[41] Wang R., Imagawa M., Honda M., Fukuhara H., Hiramatsu S. (2021). Influence of dissolved oxygen on DL-EPR behavior of SUS329J4L duplex stainless steel after convection cooling. Zair Soc. Mater. Sci. Jpn..

[42] Wang J., Yao Y., Liu Z., Tian H., Chen C., Chen L., Gonzalez-Garcia Y., Han P. (2024). The effect of N addition on the micro-structure and corrosion resistance of modified 6Mo super austenitic stainless steels during isothermal aging treatment. Ironmak. Steelmak..

[43] Nagae K., Ejiri K., Hirabayashi J., Yamamoto Y., Hatakeyama M., Sunada S. (2022). Effect of Sigma Phase on Corrosion Behavior of Duplex Stainless Steel. Mater. Trans..

