# Supplementary material for: Microstructural Evolution, Sigma Phase Morphology, and Localized Corrosion Behavior in UNS S32750 Superduplex Stainless Steel
Source: Materials (Basel). 2026 Aug 11;19(16):3413. doi: 10.3390/ma19163413 (PMC13514365; doi:10.3390/ma19163413)
Supplement: Supplementary file 1 [file materials-19-03413-s001.zip › materials-4477597-supplementary.pdf]

Article—Supplementary Material

Microstructural Evolution, Sigma Phase Morphology, and Localized Corrosion Behavior in UNS S32750 Superduplex Stainless Steel

Priscila Sousa Nilo Mendes, Patricia Sousa Nilo Mendes Raider Leoni, Elivelton Alves Ferreira and Gláucio Soares da Fonseca *

Graduate Program in Metallurgical Engineering, Federal Fluminense University, Volta Redonda 27255-125, RJ, Brazil; priscilanilomendes@gmail.com (P.S.N.M.); patysnm@gmail.com (P.S.N.M.R.L.); eliveltonalves@id.uff.br (E.A.F.)

* Correspondence: glauciofonseca@id.uff.br; Tel.: +55-24-2107-3728

The supplementary material includes an overview of the cyclic polarization behavior of all investigated conditions. While the main manuscript presents each polarization curve together with its corresponding SEM micrograph, Figure S1 enables direct visual comparison of the electrochemical responses under the different aging conditions.

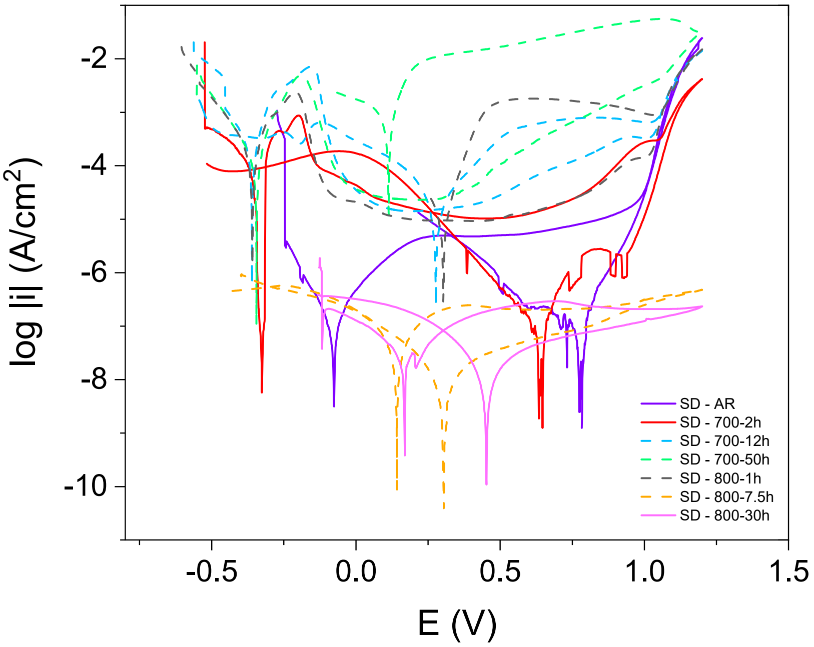

Figure S1. Comparison of the cyclic polarization curves for all investigated conditions. This figure complements the individual polarization curves presented in Figures 4–10, allowing direct comparison of the electrochemical behavior under all aging conditions. Curves with negative hysteresis are plotted using solid lines, whereas curves with positive hysteresis are plotted using dashed lines.
